# Optimization and validation of a targeted high-throughput UHPLC-MS/MS method for the analysis of multiple mycotoxins in chicken serum, egg yolk and white

**DOI:** 10.1007/s12550-025-00618-w

**Published:** 2025-12-02

**Authors:** Tadele Kabeta, Siegrid De Baere, Siska Croubels, Gunther Antonissen

**Affiliations:** 1 Faculty of Veterinary Medicine, Department of Pathobiology, Pharmacology and Zoological Medicine, Laboratory of Pharmacology and Toxicology, Ghent University, Salisburylaan 133, Ghent, 9820 Merelbeke-Melle Belgium; 2 College of Agriculture and Veterinary Medicine, School of Veterinary Medicine, Jimma University, P.O. Box 307, Jimma, Ethiopia; 3 Faculty of Veterinary Medicine, Chair Poultry Health Sciences, Ghent University, Salisburylaan 133, Ghent, 9820 Merelbeke-Melle Belgium

**Keywords:** Mycotoxins, Chicken, Eggs, Serum, UHPLC-MS/MS, Exposure assessment, Food safety

## Abstract

Mycotoxins, produced by fungi, contaminate animal feed and subsequently enter food products like eggs, posing significant health risks. This study aimed to optimize and validate a sensitive, cost-efficient, high-throughput UHPLC-MS/MS method for the qualitative analysis of 38 mycotoxins, and the quantification of 30, 29 and 29 regulated and emerging mycotoxins in chicken serum, egg yolk and egg white, respectively. Sample preparation involved liquid extraction with 0.1% formic acid in acetonitrile, followed by protein and phospholipid removal using Oasis^®^ Ostro™. This high-throughput method processed 96 samples within 4 h. Chromatographic separation was performed on an Acquity Premier BEH C18 column using 0.1% acetic acid in both water and methanol as mobile phases, with gradient elution. The MS/MS instrument employed electrospray ionization polarity switching and operated in multiple reaction monitoring mode. To enhance performance, ¹³C-labeled internal standards were utilized. In-house method validation followed European guidelines, with procedural calibration curves constructed over a range limit quantification (LOQ) up to 200 ng/ml for serum and 20 µg/kg for egg yolk/white, demonstrating good linearity (*r* ≥ 0.99). LOQ values ranged between 0.05 and 1.0 ng/ml or µg/kg for serum and egg white, respectively, and 0.05–2.5 µg/kg for egg yolk. Results for within-run and between-run accuracy and precision fell within predefined ranges. The method’s applicability was evaluated through the analysis of real serum and egg samples collected from 13 to 21 poultry farms in Ethiopia, respectively. Several mycotoxins were detected and quantified in all matrices, demonstrating the method’s value for in-vivo monitoring of mycotoxin exposure and food safety risk assessment.

**Supplementary Information:**

The online version contains supplementary material available at 10.1007/s12550-025-00618-w.

## Introduction

Mycotoxins are fungal secondary metabolites that can bioaccumulate in animal fluids, organs, and tissues (Escrivá et al. 2017). These toxic compounds are unavoidable contaminants in food and feed and have been linked to severe health issues in domestic animals and humans worldwide (Hassan et al. 2012; Razzaghi-Abyaneh et al. 2014; Tangni et al. 2020; Li et al. 2021; Osaili et al. 2022). When animals consume contaminated feed, mycotoxins can be present as parent compounds or metabolites in animal-derived food products, ultimately entering the human food chain. This poses significant health risks, ranging from severe toxicity to even death, in both humans and animals (Greco et al. 2014; Haque et al. 2020; Bozzo et al. 2023; Mafe and Büsselberg 2024; Balan et al. 2024).

To protect animals and humans from the harmful effects of these mycotoxins, maximum (guidance) levels (MLs) for some compounds have been established in certain foodstuffs (aflatoxins (AFs), ochratoxin A (OTA), patulin, deoxynivalenol (DON), zearalenone (ZEN), fumonisins (FBs) and citrinin (CIT) and products intended for animal feeding (AFs, DON, ZEN, OTA, FBs, T2 toxin (T2) and HT-2 toxin (HT-2) by the European Union (EU) (Anonymous 2006, 2023; Bozzo et al. 2023). However, no specific MLs have yet been established for mycotoxins in eggs (Frenich et al. 2011; Capriotti et al. 2012). Moreover, some emerging mycotoxins such as beauvericin (BEA), enniatins (ENNs), alternariol (AOH), alternariol monomethyl ether (AME) and tenuazonic acid (TEA) are highly prevalent in food and feed but are not yet regulated by the EU (Fraeyman et al. 2016; Meerpoel et al. 2020; Laouni et al. 2024).

Current regulatory efforts predominantly concentrate on food contamination and consumption data, which poses significant challenges in evaluating individual exposure in both animals and humans (Osteresch et al. 2017; Lauwers et al. 2019). Alternatively, mycotoxins and their relevant phase I and II metabolites in biological samples, such as plasma or serum, can serve as biomarkers, facilitating the assessment of exposure at an individual level (Lauwers et al. 2019). Serum mycotoxin concentrations provide valuable insights into exposure and are crucial for evaluating health risks in poultry. Similarly, in humans, the biomonitoring of mycotoxins in serum is supportive in identifying food chain quality issues that impact public health (Andretta et al. 2012). Serum is often preferred over plasma for analytical purposes due to its cleaner matrix, which mitigates ion suppression in ultra high-performance liquid chromatography – tandem mass spectrometry (UHPLC-MS/MS) methods, and the absence of clotting factors, that could otherwise interfere with the results (Panuwet et al. 2016).

Furthermore, co-contamination of multiple mycotoxins is frequently reported, with potential additive or synergistic effects, where the combined impact of the toxins is equal to or exceeds the sum of their individual effects, thereby posing increased risks to health and food safety (Crudo et al. 2019; Ochieng et al. 2024). This highlights the critical necessity for effective monitoring of low-concentration mycotoxins in food, feed, and biological samples through the use of sensitive, fast, and reliable methods (Escrivá et al. 2017; Lauwers et al. 2019). Immunoassay methods, relying on antigen-antibody interactions, are extensively utilized for mycotoxin detection (Tatfo Keutchatang et al. 2022). However, challenges such as cross-reactivity (causing false positive results or reduced specificity), semi-quantitative results, matrix effects, and dependence on high-quality antibodies affect their accuracy and reliability in certain applications (Li et al. 2021).

Chromatography-based methods, particularly UHPLC-MS/MS, are the gold standard in mycotoxin analysis due to their high selectivity and sensitivity (Capriotti et al. 2012; Escrivá et al. 2017; Lauwers et al. 2019). Current trends emphasize multi-class methods that minimize sample preparation for diverse matrices, including food (Yogendrarajah et al. 2013; Capriotti et al. 2014), biological samples like plasma/serum (Lauwers et al. 2019), and eggs (Frenich et al. 2011; Capriotti et al. 2012).

Simultaneous extraction of multiple mycotoxins is challenging due to their heterogeneous physico-chemical properties (Zhu RunYue et al. 2015). Sample pretreatment, especially for matrices like eggs with high fat and protein content, often becomes a bottleneck. Generic methods such as QuEChERS (quick, easy, cheap, effective, rugged, and safe) are widely used but may require additional clean-up steps to reduce severe matrix effects (ME) on UHPLC-MS/MS instruments (Frenich et al. 2011; Capriotti et al. 2014). Therefore, developing reliable sample preparation techniques and validated protocols remains critical to enable rapid and accurate mycotoxin quantification, particularly as the number of compounds concerned grows.

This study aimed to optimize and validate a cost-efficient high-throughput method to screen 38 mycotoxins and quantify 30, 29 and 29 mycotoxins in serum, egg yolk, and egg white, respectively. Using protein precipitation and phospholipid removal before UHPLC-MS/MS analysis, the method addresses challenges posed by these complex matrices and mycotoxins with diverse physico-chemical properties. To demonstrate the applicability of the method for food safety risk evaluation, real serum and egg samples from 13 to 21 poultry farms, respectively, were analyzed.

## Materials and methods

### Study samples

The egg samples (*n* = 160) were collected from 21 different layer farms in Jimma, Ethiopia, with 5 to 10 eggs sampled per farm. Serum samples (*n* = 80) were collected from chickens at 13 farms as per the owner’s permission. Blood samples were collected with a syringe from the wing vein and transferred to serum vacutainer tubes and centrifuged. The serum was stored at −20 °C until further analysis. Following collection, whole egg samples were transferred into a cooling box at 4 °C and transported within 2–3 h to the laboratory. For sampling the egg yolk and white, each egg was cracked and carefully separated into egg yolk and white. Five ml of egg yolk and egg white were placed in cryovial tubes with a 5 ml syringe and stored at − 20 °C until the moment of mycotoxin analysis.

### Materials, analytical standards and reagents

The following analytical standards were purchased from Fermentek (Jerusalem, Israel): 3-acetyl-deoxynivalenol (3-ADON), 15-acetyl-deoxynivalenol (15-ADON), AFB1, aflatoxin B2 (AFB2), aflatoxin G1 (AFG1), aflatoxin G2 (AFG2), aflatoxin M1 (AFM1), aflatoxin M2 (AFM2), AOH, AME, BEA, citrinin (CIT), DON, enniatin A (ENNA), enniatin A1 (ENNA1), enniatin B (ENNB), enniatin B1 (ENNB1), FB1, fumonisin B2 (FB2), fumonisin B3 (FB3), HT2, OTA, T2, TEA, a-zearalanol (a-ZAL), b-zearalanol (b-ZAL), a-zearalenol (a-ZEL), b-zearalenol (b-ZEL), zearalanone (ZAN), ZEN. The analytical standard of deepoxy-deoxynivalenol (DOM-1, 50 µg/ml in acetonitrile) was obtained from Merck Life Science (Hoeilaart, Belgium), whereas the standards of zearalenone-4-sulfate ammonium salt (ZEN-Sulf) and deoxynivalenol-15-glucuronide (DON-GlcA) were from TRC (Toronto, Canada). The following analytical standards were obtained as a kind gift: deoxynivalenol-sulfate (DON-Sulf), zearalenone-glucuronide (ZEN-GlcA), a-zearalenol-glucuronide (a-ZEL-GlcA), b-zearalenol-glucuronide (b-ZEL-GlcA) and dihydro-citrinone (HO-CIT).

Isotopic labeled internal standard (IS) solutions were purchased from Biopure, Romer Labs (Tulln, Austria): ^13^C_17_-AFB1, ^13^C_15_-AME, ^13^C_14_-AOH, ^13^C_13_-CIT, ^13^C_15_-DON, ^13^C_34_-FB1, ^13^C_20_-OTA, ^13^C_24_-T2, ^13^C_10_-TEA, ^13^C_18_-ZEN.

All standards were stored according to the manufacturer’s recommendations. Solvents and reagents of ULC/MS quality (acetonitrile (ACN), methanol (MeOH), formic acid (FA) and acetic acid (AA) were obtained from Biosolve (Valkenswaard, the Netherlands). Ultrapure water (H_2_O) was freshly prepared using a Milli-Q system (Merck Life Science). Oasis^®^ Ostro 96-well plates (25 mg) for protein precipitation and phospholipid removal, 2-ml square collection plates, 96-well and cap-mat square plugs silicone/PTFE treated (pre-slit) were obtained from Waters (Antwerp, Belgium).

### Preparation of standard solutions

Individual stock solutions (SS) were prepared in ACN (0.1 mg/ml: AFM1, AFM2; 1 mg/ml: AFB1, AFB2, AFG1, AFG2, DON, OTA, T2, HT2, ZEN, a-ZEL, b-ZEL, ZAN, a-ZAL, b-ZAL), or MeOH (0.1 mg/ml: AME, DON-GlcA, ZEN-Sulf; 1 mg/ml: 3-ADON, 15-ADON, AOH, BEA, CIT, ENNA, ENNA1, ENNB, ENNB1, TEA) or water/ACN (50/50, v/v) (1 mg/ml: FB1, FB2, FB3). Mixed working solutions (WS) containing 30 analytes that were determined quantitatively (WS_mix_A) were prepared at concentrations of 1000 ng/ml, 100 ng/ml, 10 ng/ml, 1 ng/ml and 0.1 ng/ml (AFB1, AFB2, AFG1, AFG2, AFM1, AFM2, 3-ADON, AOH, AME, BEA, CIT, DON, DOM-1, DON-GlcA, ENNA, ENNA1, ENNB, ENNB1, FB1, HT2, OTA, T2, TEA, ZEN, ZAN, a-ZEL, b-ZEL, a-ZAL, b-ZAL, ZEN-Sulf). In addition, a mixed WS containing 8 compounds that were only qualitatively determined (WS_mix_B) was prepared at a concentration of 100 ng/ml and 10 ng/ml (15-ADON, FB2, FB3, HO-CIT, DON-Sulf, ZEN-GlcA, a-ZEL-GlcA, b-ZEL-GlcA).

A mixed IS WS (WS_mixIS) at a concentration of 62.5 ng/ml (^13^C_15_-AME, ^13^C_14_-AOH, ^13^C_15_-DON, ^13^C_34_-FB1, ^13^C_20_-OTA, ^13^C_24_-T2, ^13^C_10_-TEA, ^13^C_18_-ZEN), 25 ng/ml (^13^C_13_-CIT) and 12.5 ng/ml (^13^C_17_-AFB1) was prepared by appropriate dilution of the SS_IS_ with ACN.

### Preparation of calibrator and quality control samples

Calibrator and quality control (QC) samples were prepared immediately before the analysis of an analytical batch of unknown samples. After thawing, 100-µl aliquots of *in-house* blank pooled serum were transferred to Eppendorf cups and spiked at analyte concentrations of 0.05, 0.1, 0.25, 0.5, 1, 2.5, 5, 10, 25, 50, 100 and 200 ng/ml to obtain calibrator samples. QC samples were spiked at concentrations of 0.5, 5 and 50 ng/ml. ACN was added up to a final sample volume of 300 µl.

Procedural calibrator samples for egg yolk and white analysis were prepared by spiking 1-g aliquots of blank pooled matrix (eggs originating from a local supermarket) at analyte concentrations of 0.05, 0.1, 0.25, 0.5, 1, 2.5, 5, 7.5, 10 and 20 µg/kg. The QC samples were spiked at concentrations of 0.25, 2.5 and 10 µg/kg. After vortex mixing (30 s), the spiked calibrator and QC samples were allowed to equilibrate at room temperature for 5 min before the start of the sample preparation procedure (see 2.5).

### Sample pretreatment of serum and egg yolk/white

Serum: To 100 µl of serum was added 25 µl of the WS_mixIS (62.5/25/12.5 ng/ml), 200 µl of ACN (only unknown samples), followed by vortex mixing for 30 s at 2500 rpm on a multi-tube vortex mixer (BenchMixer™, Benchmark, Sayreville, NY, USA). The samples were allowed to equilibrate for 5 min at room temperature, followed by the addition of 300 µl of 0.1% FA in ACN and vortex mixing for 30 s at 2500 rpm to precipitate proteins. The samples were centrifuged for 10 min at 8517 x g (Heraeus Fresco 17, Thermo Scientific, Zaventem, Belgium).

The supernatant was transferred to an Oasis^®^ Ostro 96-well plate and vacuum (15 mm Hg) was applied for 1 min. The sample filtrate was collected in a 2-ml square collector plate and evaporated to dryness under a gentle nitrogen stream. The dry residue was redissolved in 200 µl of water/MeOH (35/65, v/v) and vortex mixed for 30 Sect. (1500 rpm). A 10-µl aliquot was injected into the UHPLC-MS/MS instrument.

Egg yolk and white: To 1 g of egg yolk or egg white 25 µl of the WS_mixIS (62.5/25/12.5 ng/ml) was added, followed by vortex mixing for 30 s at 2500 rpm on a multi-tube vortex mixer. The samples were allowed to equilibrate for 5 min at room temperature, followed by the addition of 3 ml of 1% FA in ACN and vortex mixing for 5 min at 2500 rpm to precipitate proteins. The samples were further extracted for 10 min at 80 rpm on a rotary apparatus (IKA^®^ rayster digital, VWR, Leuven, Belgium) and centrifuged for 10 min at 1200 x g (Allegra X-15R Centrifuge, Beckman Coulter, Analis, Ghent, Belgium).

One ml of the supernatant was transferred to an Oasis^®^ Ostro 96-well plate and a vacuum (15 mm Hg) was applied for 1 min. The sample filtrate was collected in a 2-ml square collector plate and evaporated to dryness under a gentle nitrogen stream. The dry residue was redissolved in 200 µl of water/MeOH/FA (59.9/40/0.1, v/v/v) and vortex mixed for 30 Sect. (1500 rpm). A 5-µl aliquot was injected into the UHPLC-MS/MS instrument.

### UHPLC-MS/MS analysis of serum and egg yolk/white

The UHPLC system consisted of an Acquity Premier binary solvent manager and flow-through-needle sample manager from Waters. Chromatographic separation was achieved on an Acquity Premier BEH C18 VanGuard FIT column (50 mm x 2.1 mm i.d., dp: 1.7 μm) from Waters. The temperatures of the column oven and autosampler tray were set at 45 °C and 10 °C, respectively.

The mobile phase A consisted of 0.1% AA in water, while the mobile phase B was 0.1% AA in MeOH. A gradient elution was performed at a flow rate of 0.3 ml/min: 0–0.5 min (90% A, 10% B), 1.0 min (linear gradient to 35% B), 3.0 min (linear gradient to 60% B), 10 min (linear gradient to 95% B), 10–12.8 min (5% A, 95% B), 13.2 min (linear gradient to 10% B), 13.2–16.0 min (90% A, 10% B).

The UHPLC column effluent was interfaced with a Xevo TQ Absolute^®^ mass spectrometer, equipped with an electrospray ionization (ESI) probe operating in the positive or negative mode, depending on the mycotoxin (all from Waters). A divert valve was used, and the UHPLC effluent was directed to the mass spectrometer from 1.2 to 8.4 min.

Instrument parameters were optimized by direct infusion of working solutions of 1.0 µg/ml of all individual compounds, at a flow rate of 10 µl/min and in combination with the mobile phase (50% A, 50% B, flow rate: 200 µl/min).

The following parameters were used: capillary voltage: 3.0 kV, cone: 30 V, source offset: 30 V, desolvation temperature: 550 °C, desolvation gas: 800 l/h, cone gas: 150 l/h, nebuliser gas: 80 l/h, LM resolution 1 and 2: 2.70, HM resolution 1 and 2: 15.0 and 14.8, respectively; ion energy 1 and 2: 0.0 and 2.0, respectively; collision gas flow: 0.15 ml/min. MS/MS acquisition was performed in the multiple reaction monitoring (MRM) mode. The MRM transitions that were monitored for all analytes are shown in Table S1.

### Method validation

#### Calibration curve and range

Procedural calibration curves were prepared over a spiked concentration range between 0.05 and 200 ng/ml for serum and 0.05–20 µg/kg for egg yolk and white, respectively. Three independent calibration curves were run over several days for egg yolk and white. The slope (a), intercept (b) and correlation coefficients (*r* ≥ 0.99) were determined.

#### Accuracy and precision

Within-run accuracy and precision (repeatability) were determined by analyzing ≥ 5 pooled matrix (serum, egg yolk and egg white) samples that were spiked at the LOQ (ranging between 0.05 and 1.0 ng/ml in serum; 0.05–2.5 µg/kg in egg yolk and 0.05–1.0 µg/kg in egg white, depending on the mycotoxin) and at a low (0.5 ng/ml; 0.25 µg/kg), medium (5 ng/ml; 2.5 µg/kg) and high (50 ng/ml; 10 µg/kg) QC concentration level in the same run for serum and egg yolk/white, respectively. The between-run accuracy and precision (within-laboratory reproducibility) for egg yolk/white were determined by analyzing QC samples at the same concentration levels on three different analysis days (*n* ≥ 5 per day). For the analysis of mycotoxins in serum, cross-validation was performed. This means that between-run accuracy and precision were evaluated based on the QC samples (QClow, QCmedium and QChigh, *n* ≥ 2 per concentration level, results not shown) that were analyzed together with an analytical batch of unknown samples on three different analysis days.

For multi-mycotoxin methods in food and feed, the acceptance criteria for accuracy as specified in the EURL-MP draft guidance document were handled : −30% to + 20% (Anonymous 2024). Within-run and between-run precision were evaluated according to the same guideline by the determination of the relative standard deviation (repeatability or RSD_r_; within-laboratory reproducibility or RSD_wR_), and should be ≤ 20% (Anonymous 2024).

#### Limit of quantitation and limit of detection

The LOQ was the lowest concentration of the spiked analyte for which the method was validated with accuracy and precision that fell within the recommended ranges. The LOQ was also established as the lowest point of the calibration curve. The LOQ was determined by analyzing ≥ 5 samples spiked at a concentration level ranging between 0.05 and 1.0 ng/ml in serum; 0.05–2.5 µg/kg in egg yolk and 0.05–1.0 µg/kg in egg white (depending on the mycotoxin), on the same day. The LOD was defined as the lowest concentration that could be recognized by the detector with a signal-to-noise (S/N) ratio of ≥ 3. The LOD values were calculated using the mean S/N of the mycotoxins in serum and egg yolk/white samples spiked at the LOQ level.

#### Carry-over

The absence of carry-over was verified by analyzing the reconstitution solvent injected after the highest calibration sample. If a peak was observed in the elution zone of a mycotoxin or the ISs, it had to be below 20% of the response at the LOQ level (Anonymous 2023).

#### Selectivity and specificity

The selectivity was determined by the analysis of the blank extracted matrix. The absence of interferences at the same retention time of a mycotoxin was accepted when the response of possible interfering endogenous compounds was < 20% of the response of the corresponding analyte in the LOQ samples (Anonymous 2015). The specificity of the method was evaluated concerning other related substances (e.g., mycotoxins from the same group). The same acceptance criteria as for selectivity were handled (Anonymous 2023).

#### Extraction recovery, matrix effects and process efficiency

The RE, ME evaluated as signal suppression or enhancement (SSE), and overall, PE were assessed using three calibration curves. These were prepared with a blank matrix (egg yolk or white) spiked with mycotoxins and IS before (A) and after (B) sample treatment, and a third curve in reconstitution solvent (C). Calibration curves were plotted of analyte peak area against concentrations, using linear regression (y = ax + b, with a weighting factor of 1/x^2^; where y is peak area, a is slope, x is analyte concentration, b is intercept). The slope of each curve was used to calculate RE (slope_A_/slope_B_), SSE (slope_B_/slope_C_) and PE (slope_A_/slope_C_) (Matuszewski et al. 2003; Anonymous 2024). Additionally, curves plotting response (peak area analyte/peak area IS) versus concentration ratio (analyte/IS) were used to evaluate IS-normalized RE_N_, SSE_N_, and PE_N_.

#### Analysis of real samples to test the applicability of the method

The method’s applicability was tested by analyzing multiple mycotoxins in 80 serum samples and 160 egg white and yolk samples from chickens at different farms in Ethiopia.

## Results and discussion

A multi-mycotoxin method was optimized in chicken serum, egg yolk and white. Based on occurrence data, toxicological relevance, and the (commercial) availability of analytical standards, 38 mycotoxins and phase-I or phase-II metabolites were included.

### Sample pre-treatment

#### Serum

The protocol used was based on Lauwers et al. (Lauwers et al. 2019) for the analysis of 24 mycotoxins and major phase I and II biomarker metabolites in chicken plasma. Some minor modifications were applied regarding sample volume (100 µl versus 150 µl) and redissolution solvent, which contained a higher water content (35% versus 15%) to prevent peak fronting of early eluting compounds, such as DON and metabolites. By applying an Oasis^®^ Ostro 96-well clean-up, both phospholipids and proteins could be removed as much as possible from the serum matrix, resulting in clear extracts. This is important because it is known that chicken serum/plasma contains more phospholipids compared to other animal species (e.g. pigs) (Ferlazzo et al. 2011). Residual phospholipids in serum extracts can hamper the LC-MS/MS analysis by clogging tubing, shortening the analytical column’s lifetime and contaminating the mass spectrometer, necessitating frequent cleaning during routine sample analysis.

#### Egg yolk and white

The sample preparation protocol for egg yolk and white was based on that of De Baere et al. (De Baere et al. 2023) for the analysis of AFs in whole eggs, with some modifications. The protocol analyzes egg yolk and white separately, to evaluate the distribution of the different mycotoxins between both egg compounds, as has also been done by Osaili et al. (Osaili et al. 2022). Extracting multiple mycotoxins from the egg matrix is a real challenge due to its complexity, consisting of lipids, proteins, carbohydrates, cholesterol, fat and water soluble vitamins and minerals, carotenoids and amino acids (York et al. 2020; De Baere et al. 2023). Hence, a direct extraction method without further clean-up for the simultaneous detection of multiple mycotoxins with different physico-chemical properties and generally present at low concentrations (lower µg/kg range), might not be suitable for this complex matrix. Especially with UHPLC-MS/MS, results would be affected by significant ME if limited sample pre-treatment is applied, possibly resulting in lower sensitivity and analysis reliability.

Generally, the first step in the detection of mycotoxins from (whole) eggs is based on a simple liquid extraction using ACN (Sypecka et al. 2004; Jestoi et al. 2009; Tangni et al. 2009, 2020) or MeOH, alone or in combination with water (Tangni et al. 2009; Frenich et al. 2011; Zhu RunYue et al. 2015; Jia et al. 2016; Osaili et al. 2022; Laouni et al. 2024) and sometimes acidified with FA or AA (Osaili et al. 2022). This first extraction step also allows for the precipitation of proteins. In the current procedure, 1% FA in ACN was used (De Baere et al. 2023), because this solvent was recommended for the further clean-up step using Oasis^®^ Ostro 96-well plate. This solvent was also selected as optimal for the extraction of multiple mycotoxins from whole egg samples by Zhu Run Yue et al. (Zhu RunYue et al. 2015). To remove coextracted compounds, a further clean-up step using immunoaffinity columns (IAC) (Sypecka et al. 2004; Tangni et al. 2009; Jia et al. 2016; Bozzo et al. 2023) or SPE (Jestoi et al. 2009; Tangni et al. 2009; Zhu RunYue et al. 2015; Osaili et al. 2022) is often applied. However, for multi-mycotoxin methods, a generic clean-up step is needed to allow the extraction of as many analytes of interest as possible. The Oasis^®^ Ostro 96-well plate was chosen because this sorbent removes both remaining proteins and phospholipids from the sample extract. No QuEChERS salts have been added to remove water from the extract, as was done by other authors (Frenich et al. 2011; Capriotti et al. 2012; Zhu RunYue et al. 2015; Osaili et al. 2022; Laouni et al. 2024), since the recommended solvent composition for Oasis^®^ Ostro is H_2_O/ACN (25/75, v/v) + 1% FA. In addition, this would prolong the sample preparation process due to the need for weighing the QuEChERS salts before sample extraction. The current procedure allows for the sample pre-treatment of 96 samples within a time frame of 4 h and can therefore be considered as high-throughput.

### UHPLC-MS/MS analysis of serum, egg yolk and white

The current UHPLC-MS/MS method was optimized to analyze all included parent mycotoxins and potential phase I and II metabolites in one analytical run. All analytes were determined in the ESI polarity that gave optimal sensitivity and hence ion polarity switching (positive/negative) was used. In such a way, the total run-time could be reduced from 28 min by Lauwers et al. (Lauwers et al. 2019), who used a separate run in ESI positive (16 min) and ESI negative (12 min), to one run of 16 min. Chromatography was tested on two columns, i.e. Acquity Premier BEH C18 (50 × 2.1 mm i.d., dp: 1.7 μm) and Acquity Premier HSS-T3 (10 × 2.1 mm i.d., dp: 1.8 μm, both from Waters) using the same mobile phases and gradient composition. The HSS-T3 column gave more retention for all analytes (results not shown), but with the BEH C18 column, the peak shape for TEA was much better, although tailing was still observed (Figure S1). Chromatography on reversed-phase C18 columns was also frequently used by other authors (Capriotti et al. 2012; Zhu RunYue et al. 2015; Osaili et al. 2022) with run-times ranging between 6.5 min (Frenich et al. 2011; Laouni et al. 2024) and 35 min (Capriotti et al. 2012). Regarding chromatographic performance, TEA has been described as a difficult compound due to its metal-chelating and acidic properties, resulting in peak tailing (Fraeyman et al. 2015). Fraeyman et al. added ammonium formate to the mobile phase A (Fraeyman et al. 2015), whereas Lauwers et al. used 1% AA as a modifier (Lauwers et al. 2019) to obtain a better peak shape for TEA. However, the addition of 1% AA (Lauwers et al. 2019) was not possible for the current method due to the MaxPeak High Performance Surface (HPS) technology of the Acquity Premier UPLC system, which limits the AA concentration in the mobile phase to a maximum of 0.1%. This gave satisfactory results in the chromatographic peak shape and sensitivity for all analytes, including TEA. CIT and HO-CIT were eluted as rather broad peaks, which could be related to the higher pH of the mobile phase due to the addition of only 0.1% AA to the aqueous and organic mobile phase. This phenomenon of peak broadening of CIT and HO-CIT in function of decreasing % of organic modifier was also reported by Osteresch et al. (Osteresch et al. 2017).

MeOH was used as the organic mobile phase to enhance sensitivity for certain analytes, such as DON, ZEN, and their metabolites and FBs. This choice aligns with findings from other studies, which observed that most mycotoxins produced stronger signals in a MeOH-based mobile phase compared to ACN (Capriotti et al. 2014; Zhu RunYue et al. 2015). No baseline separation between a-ZEL and ZAN was achieved using the current chromatographic conditions. However, MRM transitions at *m/z* = 319.2 > 205.2 were specific for ZAN (Table S1), which made it possible to discriminate between ZAN and a-ZEL in real biological samples. The compounds 3-ADON and 15-ADON could not be chromatographically separated; therefore, 15-ADON was included in the qualitative WS_mix_B. This mixture also contained compounds without commercially available analytical standards (HO-CIT, DON-Sulf, ZEN-GlcA, a-ZEL-GlcA and b-ZEL-GlcA). Although the isobaric compounds FB2 and FB3 were successfully separated, they were only assessed qualitatively in this study, which focused on the quantitative analysis of FB1. Future research may explore the quantitative determination of other FBs (including FB2 and FB3) in chicken serum and eggs.

To obtain maximal sensitivity on the mass spectrometer, analytes were grouped in time windows per mycotoxin class, measuring two MRM transitions per compound for quantification and identification, respectively. Identification criteria were relative retention time (RRT, within ± 1% range of the mean RRT of the calibrator samples) and relative ion abundance ratio (qualifier ion/quantifier ion, ion ratio within ± 40% of the mean ion ratio of the calibrator samples) (Osteresch et al. 2017; Anonmyous 2021; Lehotay 2022).

### Method validation

The developed UHPLC-MS/MS method for the analysis of multiple mycotoxins in serum was based on the technique reported by Lauwers et al. (Lauwers et al. 2019), with some modifications of the chromatographic conditions. The following additional mycotoxins were included in the new method: AFB2, AFG1, AFG2, AFM2, CIT and FB1 (quantitative determination); HO-CIT, FB2 and FB3 (qualitative determination). The current method for the analysis of serum was cross-validated *in-house* in compliance with international guidelines (Anonymous 2023).

The method for the analysis of multiple mycotoxins in egg yolk and white was *in-house* developed and fully validated according to European guidelines (Anonymous 2015; Anonymous 2023; Anonymous 2024). The following parameters were evaluated: calibration curve (response function and range), within-run and between-run accuracy and precision, LOQ, limit of detection (LOD), selectivity and specificity, carry-over, ME, extraction recovery (RE) and process efficiency (PE).

#### Linearity

Procedural calibration curves were constructed to compensate for analyte loss during extraction and ME on the UHPLC-MS/MS instrument. A linear relationship between analyte response (peak area analyte/peak area IS) versus spiked analyte concentrations in the matrix was observed. A weighting factor of 1/x^2^ was applied, resulting in correlation coefficients > 0.99 for all analytes over the concentration range tested, as shown in Table 1 for egg yolk, Table S2 for serum, and Table S3 for egg white. Calibration curves in eggs reported by other authors were in the same range: 5–20 µg/kg (Capriotti et al. 2012); 0.01–22.4 µg/kg, for ENNs and BEA (Jestoi et al. 2009); 1–30 µg/kg for BEA and ENNs in lyophilized eggs (Laouni et al. 2024); LOQ – 100 µg/kg for AFs, ZEN, DON and metabolites (Zhu RunYue et al. 2015). For serum, linearity was in the same range as Lauwers et al. (Lauwers et al. 2019).

**Table 1:** Results of the evaluation of linearity (slope (a), intercept (b), correlation coefficient (r)), limit of quantification (LOQ), and limit of detection (LOD) for multiple mycotoxins in egg yolk of laying hens. Linearity was evaluated on 3 analysis days

Compound	Calibration range	a^a^	±	SD	b^a^	±	SD	r^a^	±	SD	LOQ	LOD
	(µg/kg)										(µg/kg)	(µg/kg)
AFB1	0.05 – 20.0	1.38	±	0.12	0.0126	±	0.0137	0.9970	±	0.0023	0.05	0.03
AFB2	0.10 – 20.0	2.46	±	0.20	-0.0650	±	0.0472	0.9974	±	0.0013	0.10	0.06
AFG1	0.10 – 20.0	3.89	±	0.22	-0.0296	±	0.0487	0.9984	±	0.0010	0.10	0.04
AFG2	0.05 – 20.0	2.53	±	0.16	0.0051	±	0.0132	0.9972	±	0.0016	0.05	0.02
AFM1	0.05 – 20.0	6.39	±	0.29	0.0254	±	0.0443	0.9976	±	0.0002	0.05	0.02
AFM2	2.50 – 20.0	1.35	±	0.18	0.5381	±	0.5453	0.9964	±	0.0030	2.50	1.21
AME	0.10 – 20.0	0.74	±	0.01	0.0022	±	0.0008	0.9994	±	0.0005	0.10	0.01
AOH	0.50 – 20.0	0.83	±	0.03	-0.0995	±	0.0747	0.9979	±	0.0013	0.50	0.27
CIT	1.00 – 20.0	2.47	±	0.49	0.4970	±	0.4697	0.9925	±	0.0053	1.00	0.57
DON	0.50 – 20.0	0.71	±	0.00	-0.0134	±	0.0532	0.9995	±	0.0002	0.50	0.14
DOM-1	0.50 – 20.0	0.45	±	0.01	0.0565	±	0.0403	0.9973	±	0.0012	0.50	0.27
3-ADON	2.50 – 20.0	0.07	±	0.01	0.0204	±	0.0249	0.9971	±	0.0031	2.50	1.00
DON-GlcA	1.00 – 20.0	0.06	±	0.01	-0.0036	±	0.0069	0.9976	±	0.0004	1.00	0.37
BEA	0.50 – 20.0	0.09	±	0.13	0.0067	±	0.0101	0.9919	±	0.0016	0.50	0.35
ENNA	0.10 – 20.0	0.39	±	0.38	-0.0007	±	0.0018	0.9967	±	0.0015	0.10	0.01
ENNA1	0.05 – 20.0	0.25	±	0.20	-0.0007	±	0.0011	0.9961	±	0.0015	0.05	0.01
ENNB	0.05 – 20.0	0.34	±	0.14	0.0023	±	0.0019	0.9969	±	0.0032	0.05	0.02
ENNB1	0.05 – 20.0	0.12	±	0.05	0.0007	±	0.0008	0.9962	±	0.0024	0.05	0.02
FB1	1.00 – 20.0	0.21	±	0.09	-0.0004	±	0.0200	0.9964	±	0.0019	1.00	0.29
OTA	0.05 – 20.0	1.06	±	0.03	0.0023	±	0.0029	0.9992	±	0.0004	0.05	0.01
T2	0.25 – 20.0	0.62	±	0.04	0.0131	±	0.0210	0.9960	±	0.0011	0.25	0.10
TEA	0.50 – 20.0	1.37	±	0.07	0.2816	±	0.1229	0.9976	±	0.0014	0.50	0.27
ZEN	0.05 – 20.0	0.79	±	0.01	-0.0001	±	0.0055	0.9992	±	0.0005	0.05	0.02
ZAN	0.10 – 20.0	0.73	±	0.06	0.0156	±	0.0115	0.9984	±	0.0010	0.10	0.05
a-ZEL	0.25 – 20.0	0.34	±	0.02	-0.0160	±	0.0213	0.9976	±	0.0012	0.25	0.19
b-ZEL	0.25 – 20.0	0.32	±	0.05	-0.0017	±	0.0109	0.9910	±	0.0116	0.25	0.10
a-ZAL	0.50 – 20.0	0.57	±	0.08	0.1120	±	0.0673	0.9958	±	0.0023	0.50	0.35
b-ZAL	0.10 – 20.0	0.74	±	0.12	0.0075	±	0.0072	0.9974	±	0.0020	0.10	0.03
ZEN-Sulf	0.05 – 20.0	11.11	±	2.21	-0.1215	±	0.0615	0.9968	±	0.0022	0.05	0.02

^a^Mean results (*n* = 3) ± standard deviation are shown

#### Accuracy and precision

It can be observed from Table 2 (egg yolk), Table S4 (serum) and Table S5 (egg white) that within-run precision (expressed as RSD_r_, %) and accuracy fell within the acceptance ranges as specified in the EURL-MP draft guidance document for many of the analytes of interest in the different matrices (Anonymous 2024). Results for between-run precision (RSD_wR_) and accuracy were evaluated on 3 separate analysis days for egg yolk (Table 2) and egg white (Table S5) and fell also within the predefined acceptance ranges for most compounds. These results suggest that the combination of the developed generic, high-throughput sample preparation method and the applied UHPLC-MS/MS conditions provides an effective approach for the reliable quantitative determination of mycotoxins and/or metabolites from various classes with diverse physico-chemical properties.

**Table 2:** Results of the within-run and between-run precision and accuracy evaluation for the analysis of multiple mycotoxins in chicken egg yolk

Compound	Theoretical concentration (µg/kg)	Mean concentration ± SD (µg/kg)	Precision, RSD (%)	Accuracy (%)
AFB1	0.05 ^a^	0.04 ± 0.01	18.4	−15.8
	0.05 ^b^	0.05 ± 0.01	22.8	1.1
	2.5 ^a^	2.59 ± 0.06	2.4	3.6
	2.5^b^	2.56 ± 0.11	4.3	2.4
	10.0 ^a^	10.82 ± 0.55	5.0	8.2
	10.0 ^b^	10.29 ± 0.69	6.7	2.9
AFB2	0.10 ^a^	0.10 ± 0.01	3.9	4.1
	0.10 ^b^	0.11 ± 0.01	11.6	11.0
	2.5 ^a^	2.52 ± 0.11	4.3	0.8
	2.5^b^	2.49 ± 0.15	6.1	−0.2
	10.0 ^a^	10.26 ± 0.64	6.2	2.6
	10.0 ^b^	9.72 ± 0.77	7.9	−2.8
AFG1	0.10 ^a^	0.12 ± 0.01	4.5	17.5
	0.10 ^b^	0.10 ± 0.01	13.0	0.8
	2.5 ^a^	2.49 ± 0.09	3.5	−0.6
	2.5^b^	2.51 ± 0.14	5.8	0.4
	10.0 ^a^	10.39 ± 0.77	7.4	3.9
	10.0 ^b^	10.00 ± 0.73	7.3	−0.0
AFG2	0.05 ^a^	0.04 ± 0.01	17.4	−21.3
	0.05 ^b^	0.05 ± 0.01	16.2	−6.4
	2.5 ^a^	2.47 ± 0.19	7.5	−1.2
	2.5^b^	2.54 ± 0.26	10.1	1.5
	10.0 ^a^	10.01 ± 0.67	6.7	0.1
	10.0 ^b^	9.76 ± 0.89	9.2	−2.4
AFM1	0.05 ^a^	0.04 ± 0.01	12.9	−28.6
	0.05 ^b^	0.05 ± 0.01	20.2	−9.3
	2.5 ^a^	2.43 ± 0.13	5.5	−2.9
	2.5^b^	2.53 ± 0.25	9.7	−5.8
	10.0 ^a^	9.93 ± 0.79	7.9	1.4
	10.0 ^b^	9.87 ± 0.79	8.0	−1.3
AFM2	2.5 ^a^	2.68 ± 0.33	12.2	7.2
	2.5^b^	2.43 ± 0.25	10.1	−5.8
	10.0 ^a^	10.77 ± 0.80	7.4	−2.9
	10.0 ^b^	9.64 ± 0.94	9.7	−3.6
AME	0.10 ^a^	0.10 ± 0.01	4.1	0.1
	0.10 ^b^	0.10 ± 0.00	4.2	−0.6
	2.5 ^a^	2.56 ± 0.06	2.1	2.5
	2.5^b^	2.52 ± 0.06	2.2	0.7
	10.0 ^a^	10.47 ± 0.29	2.7	4.7
	10.0 ^b^	10.17 ± 0.29	2.8	1.7
AOH	0.50 ^a^	0.57 ± 0.09	15.1	14.6
	0.50 ^b^	0.49 ± 0.09	19.3	−2.1
	2.5 ^a^	2.29 ± 0.32	14.0	−8.5
	2.5^b^	2.40 ± 0.23	9.5	−4.0
	10.0 ^a^	10.62 ± 0.43	4.0	6.2
	10.0 ^b^	10.46 ± 0.37	3.5	4.6
CIT	1.00 ^a^	0.75 ± 0.16	21.5	−25.4
	1.00 ^b^	0.95 ± 0.22	23.0	−5.3
	2.5 ^a^	1.87 ± 0.41	21.7	−25.3
	2.5^b^	2.25 ± 0.41	18.1	−10.1
	10.0 ^a^	8.04 ± 1.01	12.6	−19.6
	10.0 ^b^	9.54 ± 1.33	14.0	−4.6
DON	0.50 ^a^	0.50 ± 0.03	5.0	−0.2
	0.50 ^b^	0.48 ± 0.02	5.1	−3.1
	2.5 ^a^	2.57 ± 0.10	4.1	−1.3
	2.5^b^	2.49 ± 0.10	4.1	−0.2
	10.0 ^a^	10.29 ± 0.40	3.9	2.9
	10.0 ^b^	10.09 ± 0.48	4.8	0.9
DOM-1	0.50 ^a^	0.47 ± 0.02	3.4	−5.2
	0.50 ^b^	0.46 ± 0.04	8.2	−7.7
	2.5 ^a^	2.40 ± 0.10	4.1	−3.8
	2.5^b^	2.46 ± 0.16	6.5	−1.7
	10.0 ^a^	10.58 ± 0.78	7.3	5.8
	10.0 ^b^	10.08 ± 0.73	7.3	0.8
3-ADON	2.5 ^a^	2.31 ± 0.17	7.3	−7.6
	2.5^b^	2.45 ± 0.30	12.1	−1.9
	10.0 ^a^	10.21 ± 0.64	6.3	2.1
	10.0 ^b^	10.16 ± 0.63	6.2	1.6
DON-GlcA	1.00 ^a^	1.01 ± 0.19	18.8	0.7
	1.00 ^b^	0.99 ± 0.12	11.8	−0.6
	2.5 ^a^	2.35 ± 0.17	7.4	−6.1
	2.5^b^	2.33 ± 0.15	6.6	−6.9
	10.0 ^a^	10.12 ± 0.57	5.6	1.2
	10.0 ^b^	10.22 ± 0.59	5.8	2.2
BEA	0.50 ^a^	0.43 ± 0.07	15.7	−14.9
	0.50 ^b^	0.48 ± 0.09	19.3	−4.4
	2.5 ^a^	2.03 ± 0.49	24.2	−18.6
	2.5^b^	2.16 ± 0.47	21.6	−13.5
	10.0 ^a^	9.01 ± 0.50	5.6	−9.9
	10.0 ^b^	9.14 ± 1.37	14.9	−8.6
ENNA	0.10 ^a^	0.11 ± 0.01	7.1	9.0
	0.10 ^b^	0.09 ± 0.02	19.3	−9.6
	2.5 ^a^	1.99 ± 0.17	8.7	−20.2
	2.5^b^	2.16 ± 0.34	15.7	−13.6
	10.0 ^a^	8.66 ± 0.92	10.6	−13.5
	10.0 ^b^	8.13 ± 0.86	10.6	−18.7
ENNA1	0.05 ^a^	0.05 ± 0.01	14.0	−2.0
	0.05 ^b^	0.05 ± 0.01	11.7	−7.4
	2.5 ^a^	2.04 ± 0.12	5.9	−15.6
	2.5^b^	2.21 ± 0.27	12.1	−11.7
	10.0 ^a^	9.51 ± 0.64	6.8	−4.9
	10.0 ^b^	8.59 ± 0.97	11.3	−14.1
ENNB	0.05 ^a^	0.05 ± 0.01	7.6	4.4
	0.05 ^b^	0.05 ± 0.01	16.8	−8.3
	2.5 ^a^	2.25 ± 0.09	4.0	−9.9
	2.5^b^	2.27 ± 0.21	9.0	−9.3
	10.0 ^a^	8.97 ± 0.59	6.6	−10.3
	10.0 ^b^	9.08 ± 0.84	9.2	−9.2
ENNB1	0.05 ^a^	0.05 ± 0.01	10.2	−2.6
	0.05 ^b^	0.04 ± 0.01	19.7	−15.7
	2.5 ^a^	2.09 ± 0.08	3.92	−16.3
	2.5^b^	2.16 ± 0.32	14.9	−13.6
	10.0 ^a^	8.25 ± 0.47	5.7	−17.5
	10.0 ^b^	8.73 ± 1.01	11.5	−12.7
FB1	1.00 ^a^	0.96 ± 0.05	5.6	−3.7
	1.00 ^b^	0.89 ± 0.15	17.0	−10.6
	2.5 ^a^	2.44 ± 0.24	9.9	−2.5
	2.5^b^	2.29 ± 0.46	20.3	−8.6
	10.0 ^a^	9.59 ± 0.89	9.3	−4.1
	10.0 ^b^	9.64 ± 1.41	14.6	−3.6
OTA	0.05 ^a^	0.05 ± 0.01	2.6	2.6
	0.05 ^b^	0.05 ± 0.00	4.5	−0.1
	2.5 ^a^	2.54 ± 0.04	1.4	1.7
	2.5^b^	2.52 ± 0.08	3.3	0.9
	10.0 ^a^	10.42 ± 0.44	4.2	4.2
	10.0 ^b^	10.31 ± 0.32	3.1	3.1
T2	0.25 ^a^	0.22 ± 0.05	24.0	−11.4
	0.25 ^b^	0.24 ± 0.04	17.7	−5.3
	2.5 ^a^	2.77 ± 0.06	2.3	10.6
	2.5^b^	2.56 ± 0.22	8.4	2.5
	10.0 ^a^	10.54 ± 0.49	4.7	5.4
	10.0 ^b^	9.91 ± 0.80	8.0	−0.9
TEA	0.50 ^a^	0.44 ± 0.02	3.6	−11.4
	0.50 ^b^	0.49 ± 0.04	8.4	−1.9
	2.5 ^a^	2.35 ± 0.14	5.9	−6.1
	2.5^b^	2.46 ± 0.14	5.7	−1.5
	10.0 ^a^	10.02 ± 0.34	3.3	0.2
	10.0 ^b^	10.25 ± 0.43	4.2	2.5
ZEN	0.05 ^a^	0.05 ± 0.01	6.1	−4.0
	0.05 ^b^	0.05 ± 0.01	14.1	−3.1
	2.5 ^a^	2.49 ± 0.09	3.4	−0.3
	2.5^b^	2.54 ± 0.10	4.1	1.7
	10.0 ^a^	10.10 ± 0.43	4.3	1.0
	10.0 ^b^	10.14 ± 0.38	3.8	1.5
ZAN	0.10 ^a^	0.10 ± 0.01	8.7	−4.8
	0.10 ^b^	0.09 ± 0.01	13.7	−9.7
	2.5 ^a^	2.49 ± 0.09	3.6	−0.3
	2.5^b^	2.55 ± 0.13	5.0	2.1
	10.0 ^a^	10.14 ± 0.49	4.8	1.4
	10.0 ^b^	10.12 ± 0.56	5.5	1.3
a-ZEL	0.25 ^a^	0.23 ± 0.02	9.7	−9.1
	0.25 ^b^	0.26 ± 0.06	23.8	4.3
	2.5 ^a^	2.41 ± 0.13	5.4	−3.4
	2.5^b^	2.58 ± 0.21	8.3	3.3
	10.0 ^a^	9.11 ± 0.48	5.3	−8.9
	10.0 ^b^	10.11 ± 1.16	11.5	1.1
b-ZEL	0.25 ^a^	0.23 ± 0.02	6.4	−7.6
	0.25 ^b^	0.24 ± 0.02	8.8	−4.4
	2.5 ^a^	2.58 ± 0.10	4.1	3.1
	2.5^b^	2.66 ± 0.25	9.2	6.4
	10.0 ^a^	11.04 ± 0.74	6.7	10.4
	10.0 ^b^	10.83 ± 0.99	9.2	8.3
a-ZAL	0.50 ^a^	0.53 ± 0.06	10.9	6.3
	0.50 ^b^	0.47 ± 0.11	24.2	−5.5
	2.5 ^a^	2.56 ± 0.31	12.2	2.4
	2.5^b^	2.62 ± 0.21	8.2	4.7
	10.0 ^a^	9.99 ± 1.26	12.6	−0.1
	10.0 ^b^	10.45 ± 1.01	9.7	4.5
b-ZAL	0.10 ^a^	0.10 ± 0.01	8.6	3.0
	0.10 ^b^	0.09 ± 0.02	17.2	−9.5
	2.5 ^a^	2.62 ± 0.07	2.8	4.6
	2.5^b^	2.64 ± 0.18	6.8	5.5
	10.0 ^a^	10.87 ± 0.63	5.8	8.7
	10.0 ^b^	10.51 ± 0.87	8.3	5.2
ZEN-Sulf	0.05 ^a^	0.05 ± 0.01	5.2	−4.3
	0.05 ^b^	0.05 ± 0.00	7.8	0.6
	2.5 ^a^	2.71 ± 0.04	1.4	8.4
	2.5^b^	2.54 ± 0.22	8.8	1.6
	10.0 ^a^	11.10 ± 0.92	8.3	11.0
	10.0 ^b^	10.11 ± 1.08	10.7	1.1

^a^ Within-run accuracy and precision (*n* ≥ 5); ^b^ Between-run accuracy and precision (*n* ≥ 5, 3 analysis days); SD: standard deviation; RSD: relative standard deviation; Acceptance criteria: accuracy: −30% to + 20%, within-run precision: RSD_r_ ≤ 20%; between-run precision RSD_wR_ ≤ 20%(Anonymous 2024)

During method development, it was aimed to reach LOQ values that were as low as possible. LOQ values ranged between 0.05 and 1.0 ng/ml in chicken serum, 0.05–2.5 µg/kg in egg yolk and 0.05–1.0 µg/kg in egg white, as can be seen in Table S2, Table 1 and Table S3, respectively. The calculated LOD values ranged between 0.001 and 0.68 ng/ml (chicken serum), 0.01–1.21 µg/kg (egg yolk) and 0.01–0.66 µg/kg (egg white). When comparing LOQ and LOD values with other methods reported in the literature, it is important to discriminate between single-class and multi-class mycotoxin methods. For the latter procedures, sample preparation and LC-MS/MS analysis conditions are generally not optimal, but the best compromise to determine as many compounds as possible with acceptable sensitivity and reliability. The LOQ values obtained in the current method fell in the same range or even lower than those reported for other multi-mycotoxin methods: Capriotti et al.: 0.5–3 µg/kg for mycotoxins AFB1, OTA, BEA, ENNB, ENNB1, ENNA1 and ENNA (Capriotti et al. 2012); Frenich et al. 1–20 µg/kg in eggs for selected AFs, ENNs, BEA, CIT and OTA (Frenich et al. 2011); Laouni et al.: 0.3–0.8 µg/kg for BEA and ENNs in lyophilized eggs (Laouni et al. 2024); Sypecka et al.: 0.01 and 0.1 µg/kg for DON and ZEN, respectively, in whole eggs (Sypecka et al. 2004); Tangni et al.: LOQs for DON and DOM-1 (0.6 µg/kg), ZEN, a-ZEL and b-ZEL (3, 1.5 and 6 µg/kg, respectively), CIT (17 µg/kg) and OTA (8 µg/kg) in whole eggs (Tangni et al. 2009); Tangni et al.: LOQ for DON and its metabolites (DOM-1, 3-ADON, 1.4–3.1 µg/kg); ENNs and BEA (1.4–2.1 µg/kg); T2 and H-T2 (1.8–3.9 µg/kg); ZEN and metabolites (a-ZEL/b-ZEL, 2.0–2.1 µg/kg) (Tangni et al. 2020). LOQ ranged between 0.2 and 2 µg/kg for AFs and ZEN + metabolites and between 2 and 10 µg/kg for DON and metabolites in a multi-mycotoxin method for whole eggs (Zhu RunYue et al. 2015).

Lower LOQs were obtained for all analytes (except for DON, DON-GlcA and HT2) in serum using the current method, compared to the method for chicken plasma by Lauwers et al. (Lauwers et al. 2019). Overall, the obtained LOQ and LOD values for most mycotoxins included in the current method were sufficiently low to allow a qualitative screening (*n* = 38 analytes) and quantitative analysis of 29 or 30 mycotoxins and their relevant metabolites in egg yolk/white and serum, respectively.

#### Carry-over and selectivity

No carry-over was observed for all analytes and IS.

A blank sample was extracted and analyzed to evaluate the presence/absence of any signal at the elution time of the analytes of interest. No blank serum samples without detectable concentrations of TEA were available. In the blank egg white and yolk sample, a peak at the retention time of DOM-1 and a-ZAL (only egg yolk) could be observed in the quantifier trace. Since no peak was detected in the qualifier trace, these peaks could be considered endogenous interference. In both blank matrices, a peak was observed at the retention time of TEA. The ion ratio fell within the specified ranges for this compound and therefore, it was concluded that the blank egg was naturally contaminated with TEA. During quantification, measured TEA concentrations in real samples were corrected for the basal concentration in the “blank” matrix that was used to construct procedural calibration curves.

#### Extraction recoveries (RE)

Extraction recoveries were 68.1% to 87.8% for most analytes in egg yolk and 50.4% to 78.1% in egg white (Table S6). Generally, recoveries between 70% and 120% are considered good for food analysis (Anonymous 2024). Lower RE values were observed for CIT (2.4% and 7.4%), DON-GlcA (16.8% in egg yolk), FB1 (14.4% in egg yolk and 22.4% in egg white), and TEA (38.1% and 43.9%) in egg yolk and white, respectively. This may be attributed to their acidic physico-chemical properties, suggesting the use of a more acidic extraction solvent to improve recovery.

#### Matrix effects (signal suppression/enhancement, SSE)

Signal suppression or enhancement effects were deemed acceptable if the value ranged between 80 and 120%. Values outside this range indicate a ME. As shown in Table S6, moderate ME were observed in 17 out of 29 compounds in egg white, compared to only 6 out of 29 compounds in egg yolk. These results suggest that signal suppression is more pronounced in egg yolk due to the complexity of this matrix. Comparable or worse SSE (%) results were reported for ENNs and BEA in whole eggs with SSE ranging from 54 to 76% (Jestoi et al. 2009) and reporting SSE values between 13.7 and 30.4% (Laouni et al. 2024).

ME can be mitigated using isotopically labeled IS, as demonstrated by the calculation of IS normalized ME (SSE_N_, see Table 3). However, these standards are very expensive and therefore, one IS per class of mycotoxins was used. No isotopic labeled IS was available for BEA and ENNs. The use of ^13^C_15_-AME as IS for these analytes yielded good results for RE_N_, SSE_N_ and PE_N_. The correction for ME and losses during sample preparation was further optimized by constructing ME calibration curves for quantitative analysis (Jestoi et al. 2009). By combining ISs and procedural calibration, method performance was improved, resulting in validation results for accuracy and precision that met the acceptance criteria.

**Table 3:** Evaluation of internal standard normalized extraction recovery (RE_N_), signal suppression/enhancement (SSE_N_) and process efficiency (PE_N_) for multiple mycotoxins in egg yolk and egg white

Matrix	Egg Yolk			Egg white		
Compound	RE (%)	SSE (%)	RA (%)	RE (%)	SSE (%)	RA (%)
AFB1	94.2	109.2	102.8	97.7	100.0	97.7
AFB2	106.0	133.6	141.6	70.1	77.7	54.5
AFG1	106.4	132.6	141.0	78.6	154.0	121.1
AFG2	107.9	125.5	135.4	81.2	176.8	143.5
AFM1	102.9	122.4	125.9	101.9	101.8	103.7
AFM2	105.8	118.2	125.0	111.3	91.0	101.2
AME	89.5	108.7	97.3	105.5	91.0	96.0
AOH	89.2	104.2	93.0	117.3	79.1	92.7
CIT	65.9	104.6	68.9	121.4	92.1	111.7
DON	60.4	133.3	80.5	147.9	83.9	124.1
DOM-1	100.1	149.9	150.1	96.4	91.9	88.6
ADON	100.1	169.6	169.7	92.1	92.8	85.5
DON-GlcA	15.1	93.2	14.0	88.8	82.2	73.0
BEA	97.8	76.8	75.1	91.6	95.7	87.6
ENNA	99.9	54.5	54.5	93.0	98.5	91.5
ENNA1	94.7	95.1	90.0	93.4	93.7	87.4
ENNB	95.0	111.8	106.2	108.1	101.1	109.3
ENNB1	94.2	104.9	98.9	145.2	107.5	156.1
FB1	103.4	98.8	102.2	135.8	97.0	131.8
OTA	91.0	100.4	91.3	98.0	105.1	103.0
T2	86.7	113.0	98.0	52.1	31.1	16.2
TEA	81.1	77.4	62.7	80.4	87.2	70.2
ZEN	91.6	104.8	96.1	102.1	99.8	101.8
a-ZEL	96.6	112.5	108.7	109.0	95.5	104.1
b-ZEL	91.9	83.9	77.1	98.7	90.2	89.1
ZAN	92.4	104.8	96.8	97.6	98.3	96.0
a-ZAL	91.3	99.2	90.6	102.7	92.5	95.0
b-ZAL	89.2	97.0	86.5	99.6	92.1	91.8
ZEN-Sulf	94.3	113.2	106.7	87.1	104.5	91.0

### Sample analysis

**Fig. 1:**
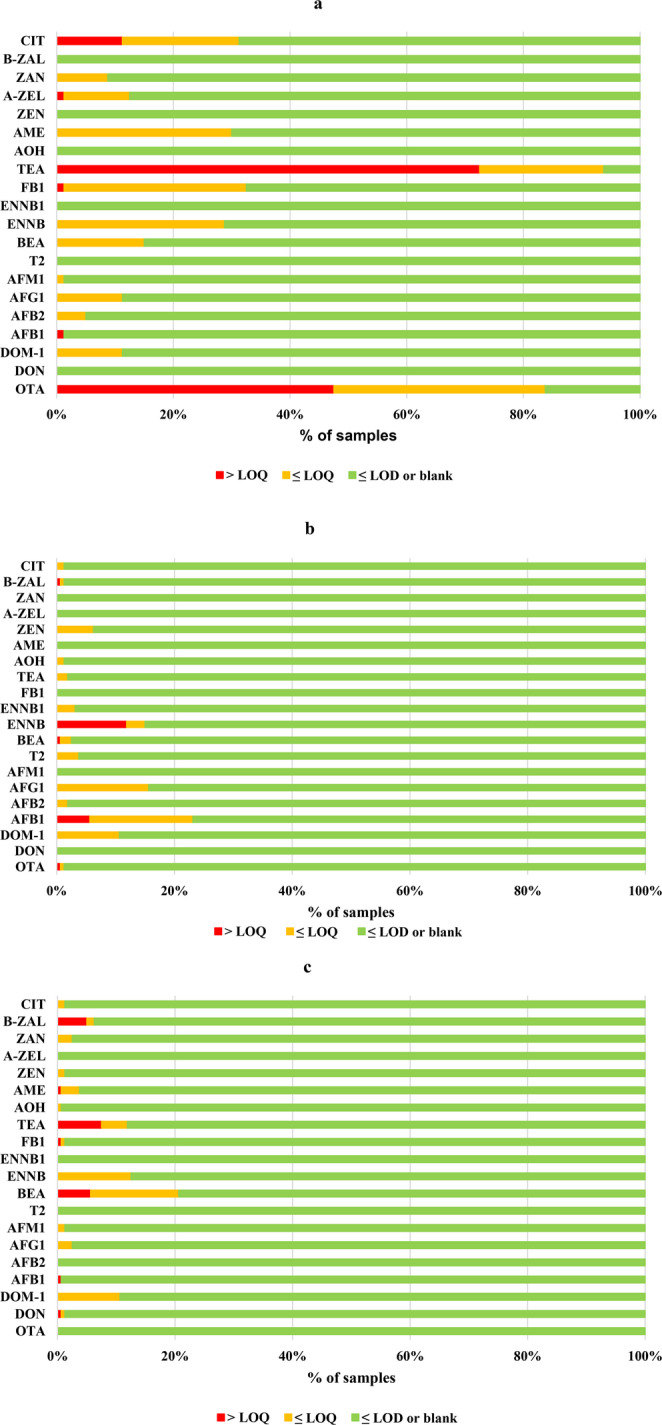
Occurrence of mycotoxins in (**a**) serum, (**b**) egg yolk and (**c**) egg white samples

Figure 1 shows the percentage of serum (a), egg yolk (b) and egg white (c) samples collected from chickens raised on 13 (serum) and 21 (eggs) poultry farms in Jimma, Ethiopia, that were screened for the presence of mycotoxins. The results are categorized by sample type and indicate whether the samples were: blank (green bar = □) or had mycotoxin concentrations between LOD and LOQ or samples ≤ limit of quantification (LOQ), or samples exceeded the LOQ (red bar = □) depending on concentration and positivity of samples for each compound.

An overview of mean mycotoxin concentrations (± SD) in serum (*n* = 80), egg yolk (*n* = 160) or egg white (*n* = 160) of chickens that were reared at 13 (serum) or 21 (eggs) poultry production farms in Ethiopia, is given in Table S7. Various mycotoxins (CIT, ZAN, a-ZEL, AME, TEA, FB1, ENNB, BEA, AFs, DOM-1 and OTA) were detected in serum samples at concentrations above the LOD, indicating that serum is a good matrix for biomonitoring animals’ exposure to mycotoxins (Lauwers et al. 2019). Concentrations above the LOQ were most frequently observed for TEA (mean conc.: 1.25 ± 1.81 ng/ml) and OTA (mean conc.: 0.01 ± 0.12 ng/ml). This observation is consistent with previous toxicokinetic studies demonstrating that TEA and OTA are fully absorbed following oral administration in broiler chickens (Fraeyman et al. 2015; Devreese et al. 2018). Additionally, the elimination half-life (T1/2el) of TEA was found to be longer in chickens compared to pigs.

AFB1 was detected in only one serum sample at a concentration > LOQ (conc.: 0.17 ng/ml). Furthermore, only trace amounts of aflatoxins (concentrations < LOQ) were measured in the serum samples. These findings align with a previous study (Ochieng et al. 2024) where AFB1 concentrations in the plasma of laying hens ranged from the LOQ to 0.063 ng/ml.

Detecting multiple mycotoxins in egg samples is challenging due to the varying polarities of target analytes and their differential distribution between yolk and albumen. As a result, some researchers (Capriotti et al. 2012) prefer to analyze whole eggs rather than separating the yolk and white. The newly developed protocol confirmed the presence of multiple mycotoxins in both egg yolk and white at trace levels (concentrations below the LOQ), specifically in 86 out of 160 egg yolk samples and 61 out of 160 egg white samples (Fig. 1b and c, respectively). For a few mycotoxins, concentrations above the LOQ were detected in egg yolk (ENNB, BEA, AFB1, OTA, b-ZAL) and egg white (BEA, AFB1, DON, FB1, TEA, AME, b-ZAL). However, the prevalence of mycotoxin concentrations above the LOQ was low, ranging from 0.6% to 11.9% in egg yolk and 0.6% to 7.5% in egg white.

Using the developed method, concentrations of ENNB and BEA above the LOQ were detected in some egg yolks (ENNB: mean conc. 0.13 ± 0.05 µg/kg; max. conc. 0.24 µg/kg; BEA: mean conc. 0.01 ± 0.06 µg/kg; max. conc. 0.53 µg/kg). In egg white, only BEA was determined at concentrations above the LOQ in some samples (mean conc. 0.06 ± 0.01 µg/kg; max. conc. 0.07 µg/kg). No ENNA or ENNA1 were detected in any of the egg yolk or white samples. Similarly, Jestoi et al. reported that ENNA and A1 were not found in any whole egg samples from a national residue monitoring program but were present in a few samples (< 2%) obtained from local grocery markets in Finland (Jestoi et al. 2009). Furthermore, no ENNs and BEA were detected in chicken eggs randomly collected from different layer farms in Algeria (Laouni et al. 2024). Santini et al. suggested that the uptake of ENNs and BEA in the gastrointestinal tract may be impaired, or these compounds may be quickly eliminated from the body through metabolization and excretion, which could explain the low concentrations of these compounds in biological matrices, such as egg yolk and white samples (Santini et al. 2012).

Trace levels (< LOQ) of AFs were measured in both egg yolk and white, with AFB1 being the only aflatoxin detected at concentrations above the LOQ in some samples (egg white: one sample, conc. 0.08 µg/kg; egg yolk: mean conc. 0.07 ± 0.01 µg/kg; max. conc. 0.10 µg/kg). These results align with findings from other researchers, reporting AFB1 concentrations up to 0.04 µg/kg (Ochieng et al. 2024), between 0.05 and 0.16 µg/kg (Oliveira et al. 2000) or mean conc. of 0.54 ± 0.43 µg/kg in Jordan chicken egg samples (Osaili et al. 2022) and 0.8 ± 1.1 µg/kg in bulk eggs in Cameroon (Tatfo Keutchatang et al. 2022). In all these studies, AFB1 residues in eggs remained below the European Union’s maximum tolerable level of 2 µg/kg for AFB1 in human food products (Tatfo Keutchatang et al. 2022).

DON was detected in only one egg white sample at a concentration > LOQ (0.28 µg/kg), whereas no DON was observed in egg yolk samples. This finding is consistent with a previous study from Jordan, where no DON was detected in 250 egg samples collected from marketplaces (Osaili et al. 2022). Trace levels of DOM-1 were found in 10.6% of both egg yolk and white samples.

ZEN and its in-vivo phase-I metabolite ZAN were determined at concentrations below the LOQ in both egg whites and yolks, whereas a-ZEL, b-ZEL and a-ZAL were not detected. b-ZAL concentrations above the LOQ were observed in one egg yolk (conc. 0.26 µg/kg) and some egg white (mean conc. 0.09 ± 0.05 µg/kg; max. conc 0.20 µg/kg) samples. Similarly, samples of backyard chickens in Belgium did not contain quantifiable levels of ZEN or its metabolites (a-ZEL/b-ZEL) (Tangni et al. 2009).

T2 was detected at low concentrations (< LOQ) in egg yolk samples, which corresponds with the generally low transmission rates of T2 from contaminated feed to chicken eggs, ranging from 0.012% to 0.13% (Jestoi et al. 2009). OTA was observed in a few egg yolk samples at trace levels, with only one sample having a concentration > LOQ (conc. 0.06 µg/kg). No concentrations of CIT above the LOQ were determined in all egg yolk and white samples.

The above results highlight the effectiveness of the developed method for biomonitoring mycotoxin exposure through serum analysis and for screening and quantifying these toxins in animal products intended for human consumption, such as eggs.

In conclusion, the developed and validated multi-mycotoxin method enabled the qualitative screening of 38 mycotoxins, including aflatoxins, ochratoxin A, *Fusarium* mycotoxins and emerging mycotoxins (*Alternaria* toxins and enniatins) in chicken serum, egg yolk and egg white. Furthermore, the method facilitated the quantitative analysis of 29 (egg yolk/white) or 30 (serum) mycotoxins and relevant in-vivo phase-I or phase-II metabolites for the first time. The sample preparation procedure was simple and straightforward, with an UHPLC-MS/MS run of 16 min, allowing the extraction and analysis of approximately 96 samples per day. The method’s applicability was assessed by analyzing 80 serum samples and 160 egg white and yolk samples from chickens of 38 Ethiopian farms, demonstrating its suitability for exposure biomonitoring, residue analysis, food safety monitoring and risk assessment.

## Supplementary information

Below is the link to the electronic supplementary material.

Supplementary File 1 (DOCX 475 KB)

## Data Availability

Data will be made available on request.

## Abbreviations

3/15-ADON: 3/15-acetyl-deoxynivalenol
AA: Acetic acid
ACN: Acetonitrile
AF: Aflatoxin
ALT: Altenuene
AME: Alternariol monomethyl ether
AOH: Alternariol
a/b-ZAL: Alfa/beta-zearalanol
a/b-ZEL: Alfa/beta-zearalenol
BEA: Beauvericin
CCa: Decision limit
CIT: Citrinin
DAS: diacetoxyscirpenol
DOM-1: Depoxy-deoxynivalenol
DON: Deoxynivalenol
ENN: Enniatin
ESI: Electrospray ionization
EU: European Union
FA: Formic acid
FB: Fumonisin
F-X: Fusarenon-X
GlcA: Glucuronide conjugate
HM: High mass
HO-CIT: Dihydro-citrinone
HPS: High Performance Surface
HT2: HT2-toxin
IAC: Immunoaffinity column
IS: Internal standard
LC: Liquid chromatography
LM: Low mass
ME: Matrix effects
LOD: Limit of detection
LOQ: Limit of quantification
MeOH: Methanol
ML: Maximum level
MRM: Multiple reaction monitoring
MS/MS: Tandem mass spectrometry
m/z: Mass-to-charge ratio
NEO: Neosolaniol
NIV: Nivalenol
OTA: Ochratoxin A
PE: Process efficiency
QC: Quality control
QuEChERS: Quick, Easy, Cheap, Effective, Rugged and Safe
r: Correlation coefficient
RE: Extraction recovery
RRT: Relative retention time
RSD: Relative standard deviation
ROQ-C: Roquefortine-C
SD: Standard deviation
S/N: Signal-to-noise ratio
SPE: Solid-phase extraction
SS: Stock solution
SSE: Signal suppression or enhancement
STERIG: Sterigmatocystin
Sulf: Sulfate conjugate
T1/2el: Elimination half-life
TEA: Tenuazonic acid
T2: T2-toxin
UHPLC: Ultra high-performance liquid chromatography
WS: Working solution
ZAN: Zearalenone
ZEN: Zearalenone
