# Supplementary material for: Optimization and validation of a targeted high-throughput UHPLC-MS/MS method for the analysis of multiple mycotoxins in chicken serum, egg yolk and white
Source: Mycotoxin Res. 2025 Dec 2;42(1):8. doi: 10.1007/s12550-025-00618-w (PMC12672711; doi:10.1007/s12550-025-00618-w)
Supplement: Supplementary file 1 — (DOCX 475 KB) [file 12550_2025_618_MOESM1_ESM.docx]

**SUPPLEMENTARY MATERIAL**

**Optimization and validation of a targeted high-throughput UHPLC-MS/MS method for the analysis of multiple mycotoxins in chicken serum, egg yolk and white**

Tadele Kabeta^§,*1,2^, Siegrid De Baere^§,*,1^, Siska Croubels^1^ and Gunther Antonissen^1,3^

^1^Ghent University, Faculty of Veterinary Medicine, Department of Pathobiology, Pharmacology and Zoological Medicine, Laboratory of Pharmacology and Toxicology, Salisburylaan 133, 9820 Merelbeke-Melle, Belgium; ^1^[Tadelekabeta.yadessa@ugent.be](mailto:Tadelekabeta.yadessa@ugent.be) (T.K.); [Siegrid.Debaere@ugent.be](mailto:Siegrid.Debaere@ugent.be) (S.D.B.); [Siska.Croubels@UGent.be](mailto:Siska.Croubels@UGent.be) (S.C.); [Gunther.Antonissen@ugent.be](mailto:Gunther.Antonissen@ugent.be) (G.A.)

^2^Jimma University, College of Agriculture and Veterinary Medicine, School of Veterinary Medicine P.O. Box 307, Jimma, Ethiopia;

^3^Ghent University, Faculty of Veterinary Medicine, Chair Poultry Health Sciences, Salisburylaan 133, 9820 Merelbeke-Melle, Belgium.

**^§^Shared first authors**

^*^**Corresponding authors**: ^1^[Siegrid.Debaere@ugent.be](mailto:Siegrid.Debaere@ugent.be); ^1,2^[Tadele.kabeta@ju.edu.et](mailto:Tadele.kabeta@ju.edu.et)

**Table S1.** MRM transitions and MS/MS parameters for analysis of mycotoxins in chicken serum, egg yolk and white

| **Analyte** | **ESI mode** | **Precursor**  **ion**  **(*m/z*)^a^** | **Product**  **ions**  **(*m/z*)** | **CE^b^**  **(eV)** | **Cone**  **(V)** | **Retention**  **time**  **(min)** | **Internal standard** | **Quan/Qual^d^** |
| --- | --- | --- | --- | --- | --- | --- | --- | --- |
| ^13^C_17_-AFB1 | + | 330.2 | 301.2^c^  255.1 | 24  38 | 30  30 | 2.74 | / | / |
| AFB1 | + | 313.2 | 241.1^c^  285.1 | 36  26 | 30  30 | 2.74 | ^13^C_17_-AFB1 | Quan |
| AFB2 | + | 315.2 | 287.2^c^  259.1 | 26  28 | 30  30 | 2.59 | ^13^C_17_-AFB1 | Quan |
| AFG1 | + | 329.2 | 243.1^c^  311.1 | 28  22 | 30  30 | 2.46 | ^13^C_17_-AFB1 | Quan |
| AFG2 | + | 331.2 | 245.1^c^  313.1 | 30  24 | 30  30 | 2.32 | ^13^C_17_-AFB1 | Quan |
| AFM1 | + | 329.2 | 273.1^c^  229.1 | 26  40 | 30  30 | 2.34 | ^13^C_17_-AFB1 | Quan |
| AFM2 | + | 331.3 | 313.2^c^  285.1 | 16  24 | 30  30 | 2.19 | ^13^C_17_-AFB1 | Quan |
| ^13^C_15_-AME | - | 286.1 | 270.1^c^  241.1 | 22  28 | 30  30 | 4.34 | / | / |
| AME | - | 271.1 | 256.0^c^  228.1 | 22  28 | 30  30 | 4.34 | ^13^C_15_-AME | Quan |
| ^13^C_14_-AOH | - | 271.1 | 226.0^c^  168.0 | 22  30 | 30  30 | 3.25 | / | / |
| AOH | - | 257.1 | 213.1^c^  185.0 | 24  26 | 30  30 | 3.25 | ^13^C_14_-AOH | Quan |
| ^13^C_13_-CIT | - | 294.2 | 262.1^c^  217.2 | 16  24 | 30  30 | 7.10 | / | / |
| CIT | - | 281.1 | 249.1^c^  205.1 | 18  22 | 30  30 | 7.11 | ^13^C_13_-CIT | Quan |
| OH-CIT | - | 265.1 | 221.1^c^  177.1 | 17  26 | 30  30 | 6.49 | ^13^C_13_-CIT | Qual |
| ^13^C_15_-DON | + | 312.3 | 263.2^c^  245.2 | 12  12 | 30  30 | 1.60 | / | / |
| DON | + | 297.2 | 249.1^c^  203.1 | 12  15 | 30  30 | 1.60 | ^13^C_15_-DON | Quan |
| DOM-1 | + | 281.2 | 215.1 ^c^  233.1 | 11  11 | 30  30 | 1.89 | ^13^C_15_-DON | Quan |
| ADON | + | 339.3 | 137.2 ^c^  321.3 | 9  9 | 30  30 | 2.18 | ^13^C_15_-DON | Quan |
| DON-GlcA | - | 471.3 | 193.0 ^c^  265.1 | 26  26 | 30  30 | 1.59 | ^13^C_15_-DON | Quan |
| DON-Sulf | + | 375.1 | 345.1 ^c^  97.0 | 26  34 | 30  30 | 2.49 | ^13^C_15_-DON | Qual |
| BEA | + | 784.7 | 244.1^c^  262.2 | 24  26 | 50  50 | 7.40 | ^13^C_15_-AME | Quan |
| ENNA | + | 704.7 | 350.3^c^  232.2 | 52  58 | 50  50 | 7.98 | ^13^C_15_-AME | Quan |
| ENNA1 | + | 668.7 | 210.2^c^  228.2 | 24  24 | 50  50 | 7.64 | ^13^C_15_-AME | Quan |
| ENNB | + | 640.6 | 214.2^c^  196.1 | 26  26 | 50  50 | 6.90 | ^13^C_15_-AME | Quan |
| ENNB1 | + | 671.7 | 196.1^c^  210.2 | 30  30 | 50  50 | 7.29 | ^13^C_15_-AME | Quan |
| ^13^C_34_-FB1 | + | 756.7 | 356.4^c^  374.5 | 40  35 | 30  30 | 3.54 | / | / |
| FB1 | + | 722.4 | 334.4^c^  352.4 | 37  40 | 30  30 | 3.55 | ^13^C_34_-FB1 | Quan |
| FB2 | + | 706.3 | 336.4^c^  354.4 | 42  35 | 30  30 | 4.44 | ^13^C_34_-FB1 | Qual |
| FB3 | + | 706.3 | 336.4^c^  354.4 | 42  35 | 30  30 | 4.08 | ^13^C_34_-FB1 | Qual |
| ^13^C_20_-OTA | + | 424.2 | 250.1^c^  377.2 | 24  15 | 30  30 | 5.12 | / | / |
| OTA | + | 404.2 | 239.1^c^  221.0 | 25  34 | 30  30 | 5.12 | ^13^C_20_-OTA | Quan |
| ^13^C_24_-T2 | + | 508.5 | 229.2^c^  198.1 | 22  22 | 30  30 | 3.86 | / | / |
| T2 | + | 484.3 | 305.2^c^  215.2 | 14  20 | 30  30 | 3.86 | ^13^C_24_-T2 | Quan |
| HT2 | + | 442.3 | 215.1^c^  263.1 | 12  12 | 30  30 | 3.48 | ^13^C_24_-T2 | Qual |
| ^13^C_10_-TEA | - | 206.1 | 145.0^c^  117.0 | 20  24 | 30  30 | 3.16 | / | / |
| TEA | - | 196.1 | 112.0^c^  139.0 | 25  20 | 30  30 | 3.16 | ^13^C_10_-TeA | Quan |
| ^13^C_18_-ZEN | - | 335.3 | 185.1^c^  169.0 | 24  32 | 30  30 | 4.09 | / | / |
| ZEN | - | 317.2 | 131.1^c^  175.0 | 30  24 | 30  30 | 4.09 | ^13^C_18_-ZEN | Quan |
| ZAN | - | 319.2 | 205.2^c^  275.2 | 20  24 | 30  30 | 4.00 | ^13^C_18_-ZEN | Quan |
| a-ZEL | - | 319.2 | 275.2^c^  301.2 | 20  24 | 30  30 | 3.92 | ^13^C_18_-ZEN | Quan |
| b-ZEL | - | 319.2 | 275.2^c^  301.2 | 20  24 | 30  30 | 3.57 | ^13^C_18_-ZEN | Quan |
| a-ZAL | - | 321.2 | 277.2^c^  303.2 | 22  22 | 30  30 | 3.82 | ^13^C_18_-ZEN | Quan |
| b-ZAL | - | 321.2 | 277.2^c^  303.2 | 22  22 | 30  30 | 3.44 | ^13^C_18_-ZEN | Quan |
| ZEN-Sulf | - | 397.3 | 317.2^c^  175.1 | 24  35 | 30  30 | 6.42 | ^13^C_18_-ZEN | Quan |
| ZEN-GlcA | - | 493.3 | 317.2^c^  113.0 | 26  20 | 30  30 | 4.02 | ^13^C_18_-ZEN | Qual |
| a-ZEL-GlcA | - | 495.3 | 319.2^c^  175.0 | 28  18 | 30  30 | 3.76 | ^13^C_18_-ZEN | Qual |
| b-ZEL-GlcA | - | 495.3 | 319.2^c^  113.0 | 28  22 | 30  30 | 3.30 | 13C-ZEN | Qual |

^a^ m/z = mass to charge ratio, ^b^ CE = collision energy, ^c^ ion used for quantification, ^d^Quan/Qual = quantitative or qualitative analysis

**Table S2.** Results of the evaluation of linearity (slope (a), intercept (b), correlation coefficient (r)), limit of quantification (LOQ), and limit of detection (LOD) for multiple mycotoxins in serum chickens. Linearity was evaluated on one analysis day.

| **Component** | **Calibration Range** | **a** | **b** | **r** | **LOQ** | **LOD** |
| --- | --- | --- | --- | --- | --- | --- |
|  | **(ng/mL)** |  |  |  | **(ng/mL)** | **(ng/mL)** |
| AFB1 | 0.10 – 200.0 | 0.13 | 0.0025 | 0.9988 | 0.10 | 0.03 |
| AFB2 | 0.10 – 200.0 | 0.32 | -0.0022 | 0.9976 | 0.10 | 0.01 |
| AFG1 | 0.25 – 200.0 | 0.48 | -0.0484 | 0.9987 | 0.25 | 0.01 |
| AFG2 | 0.25 – 200.0 | 0.19 | -0.0123 | 0.9975 | 0.25 | 0.04 |
| AFM1 | 0.05 – 200.0 | 0.61 | -0.0002 | 0.9988 | 0.05 | 0.01 |
| AFM2 | 0.50 – 200.0 | 0.10 | 0.0430 | 0.9972 | 0.50 | 0.10 |
| AME | 0.05 – 200.0 | 0.07 | 0.0019 | 0.9996 | 0.05 | 0.001 |
| AOH | 0.25 – 200.0 | 0.08 | 0.0042 | 0.9986 | 0.25 | 0.03 |
| CIT | 0.1 – 200.0 | 0.48 | 0.0323 | 0.9981 | 0.10 | 0.01 |
| DON | 0.50 – 200.0 | 0.09 | -0.1198 | 0.9979 | 2.50 | 0.42 |
| DOM-1 | 0.50 – 200.0 | 0.06 | -0.0098 | 0.9984 | 0.50 | 0.12 |
| 3-ADON | 0.50 – 200.0 | 0.01 | 0.0205 | 0.9950 | 0.50 | 0.09 |
| DON-GlcA | 1.00 – 200.0 | 0.01 | -0.0013 | 0.9979 | 1.00 | 0.30 |
| BEA | 0.05 – 200.0 | 0.03 | -0.0002 | 0.9987 | 0.05 | 0.01 |
| ENNA | 0.05 – 200.0 | 0.07 | 0.0001 | 0.9992 | 0.05 | 0.002 |
| ENNA1 | 0.05 – 200.0 | 0.04 | 0.0002 | 0.9992 | 0.05 | 0.01 |
| ENNB | 0.05 – 200.0 | 0.04 | 0.0000 | 0.9979 | 0.05 | 0.004 |
| ENNB1 | 0.05 – 200.0 | 0.03 | -0.0001 | 0.9989 | 0.05 | 0.01 |
| FB1 | 0.25 – 200.0 | 0.01 | 0.0002 | 0.9911 | 0.25 | 0.05 |
| OTA | 0.05 – 200.0 | 0.10 | 0.0001 | 0.9996 | 0.05 | 0.001 |
| T2 | 0.50 – 200.0 | 0.07 | -0.0005 | 0.9987 | 0.50 | 0.20 |
| HT2 | 1.0 – 200.0 | 0.01 | -0.0045 | 0.9982 | 1.00 | 0.68 |
| TEA | 0.25 – 200.0 | 0.12 | 0.0320 | 0.9977 | 0.25 | 0.04 |
| ZEN | 0.05 – 200.0 | 0.07 | 0.0402 | 0.9986 | 0.05 | 0.01 |
| ZAN | 0.25 – 200.0 | 0.09 | 0.0122 | 0.9974 | 0.25 | 0.03 |
| a-ZEL | 0.25 – 200.0 | 0.05 | 0.0106 | 0.9972 | 0.25 | 0.03 |
| b-ZEL | 0.25 – 200.0 | 0.12 | 0.0199 | 0.9955 | 0.25 | 0.04 |
| a-ZAL | 0.25 – 200.0 | 0.10 | 0.0021 | 0.9978 | 0.25 | 0.05 |
| b-ZAL | 0.25 – 200.0 | 0.29 | -0.0058 | 0.9975 | 0.25 | 0.08 |
| ZEN-Sulf | 0.25 – 200.0 | 2.74 | 26.1157 | 0.9945 | 0.25 | 0.05 |

**Table S3.** Results of the evaluation of linearity (slope (a), intercept (b), correlation coefficient (r)), limit of quantification (LOQ), and limit of detection (LOD) for multiple mycotoxins in egg white of laying hens. Linearity was evaluated on 3 analysis days.

| **Component** | **Calibration Range**  **(µg/kg)** | **a^a^** | ± | **SD** | **b^a^** | ± | **SD** | **r^a^** | ± | **SD** | **LOQ (µg/kg)** | **LOD (µg/kg)** |
| --- | --- | --- | --- | --- | --- | --- | --- | --- | --- | --- | --- | --- |
| AFB1 | 0.05 – 20.0 | 1.30 | ± | 0.02 | 0.0126 | ± | 0.0140 | 0.9980 | ± | 0.0013 | 0.05 | 0.03 |
| AFB2 | 0.05 – 20.0 | 2.43 | ± | 0.31 | -0.0203 | ± | 0.0218 | 0.9980 | ± | 0.0006 | 0.05 | 0.03 |
| AFG1 | 0.05 – 20.0 | 3.52 | ± | 0.21 | -0.0392 | ± | 0.0301 | 0.9986 | ± | 0.0005 | 0.05 | 0.03 |
| AFG2 | 0.05 – 20.0 | 2.32 | ± | 0.27 | 0.0003 | ± | 0.0228 | 0.9985 | ± | 0.0001 | 0.05 | 0.03 |
| AFM1 | 0.05 – 20.0 | 6.31 | ± | 0.49 | -0.0192 | ± | 0.0220 | 0.9981 | ± | 0.0009 | 0.05 | 0.01 |
| AFM2 | 1.0 – 20.0 | 1.58 | ± | 0.25 | 0.4368 | ± | 0.1521 | 0.9968 | ± | 0.0023 | 1.00 | 0.29 |
| AME | 0.05 – 20.0 | 0.73 | ± | 0.02 | 0.0005 | ± | 0.0010 | 0.9991 | ± | 0.0007 | 0.05 | 0.01 |
| AOH | 0.50 – 20.0 | 0.79 | ± | 0.09 | -0.0602 | ± | 0.0547 | 0.9979 | ± | 0.0006 | 0.50 | 0.21 |
| CIT | 0.25 – 20.0 | 3.66 | ± | 0.37 | -0.0478 | ± | 0.0488 | 0.9990 | ± | 0.0005 | 0.25 | 0.04 |
| DON | 0.25 – 20.0 | 0.76 | ± | 0.01 | 0.0364 | ± | 0.0138 | 0.9986 | ± | 0.0008 | 0.25 | 0.06 |
| DOM-1 | 0.50 – 20.0 | 0.44 | ± | 0.02 | 0.0581 | ± | 0.0563 | 0.9973 | ± | 0.0020 | 0.50 | 0.39 |
| 3-ADON | 1.00 – 20.0 | 0.06 | ± | 0.00 | 0.0020 | ± | 0.0117 | 0.9979 | ± | 0.0009 | 1.00 | 0.60 |
| DON-GlcA | 1.00 – 20.0 | 0.12 | ± | 0.02 | -0.0129 | ± | 0.0166 | 0.9976 | ± | 0.0013 | 1.00 | 0.41 |
| BEA | 0.05 – 20.0 | 0.31 | ± | 0.04 | 0.0008 | ± | 0.0033 | 0.9983 | ± | 0.0008 | 0.05 | 0.03 |
| ENNA | 0.05 – 20.0 | 1.00 | ± | 0.08 | 0.0059 | ± | 0.0071 | 0.9983 | ± | 0.0011 | 0.05 | 0.02 |
| ENNA1 | 0.05 – 20.0 | 0.47 | ± | 0.03 | -0.0019 | ± | 0.0011 | 0.9983 | ± | 0.0011 | 0.05 | 0.01 |
| ENNB | 0.05 – 20.0 | 0.41 | ± | 0.03 | -0.0021 | ± | 0.0016 | 0.9974 | ± | 0.0021 | 0.05 | 0.01 |
| ENNB1 | 0.05 – 20.0 | 0.24 | ± | 0.05 | -0.0009 | ± | 0.0004 | 0.9982 | ± | 0.0012 | 0.05 | 0.01 |
| FB1 | 0.25 – 20.0 | 0.15 | ± | 0.03 | 0.0018 | ± | 0.0075 | 0.9976 | ± | 0.0004 | 0.25 | 0.15 |
| OTA | 0.05 – 20.0 | 0.96 | ± | 0.06 | 0.0012 | ± | 0.0019 | 0.9996 | ± | 0.0002 | 0.05 | 0.02 |
| T2 | 1.0 – 20.0 | 0.58 | ± | 0.09 | 0.0534 | ± | 0.1039 | 0.9974 | ± | 0.0004 | 1.00 | 0.54 |
| TEA | 1.0 – 20.0 | 1.20 | ± | 0.14 | 1.3054 | ± | 0.2655 | 0.9983 | ± | 0.0008 | 1.00 | 0.66 |
| ZEN | 0.05 – 20.0 | 0.74 | ± | 0.01 | -0.0008 | ± | 0.0023 | 0.9988 | ± | 0.0011 | 0.05 | 0.01 |
| ZAN | 0.05 – 20.0 | 0.69 | ± | 0.05 | -0.0007 | ± | 0.0003 | 0.9979 | ± | 0.0003 | 0.05 | 0.01 |
| a-ZEL | 0.10 – 20.0 | 0.30 | ± | 0.02 | 0.0043 | ± | 0.0042 | 0.9979 | ± | 0.0015 | 0.10 | 0.04 |
| b-ZEL | 0.25 – 20.0 | 0.27 | ± | 0.02 | 0.0034 | ± | 0.0065 | 0.9985 | ± | 0.0004 | 0.25 | 0.07 |
| a-ZAL | 0.50 – 20.0 | 0.48 | ± | 0.04 | 0.0989 | ± | 0.0662 | 0.9953 | ± | 0.0038 | 0.25 | 0.41 |
| b-ZAL | 0.05 – 20.0 | 0.64 | ± | 0.06 | 0.0047 | ± | 0.0016 | 0.9989 | ± | 0.0002 | 0.05 | 0.01 |
| ZEN-Sulf | 0.25 – 20.0 | 7.66 | ± | 2.32 | -0.0259 | ± | 0.0211 | 0.9981 | ± | 0.0012 | 0.25 | 0.01 |

Note: ^a^Mean results (n = 3) ± standard deviation are shown

**Table S4.** Results of the within-run precision and accuracy evaluation for the quality control samples of the analysis batch of multiple mycotoxins in chicken serum.

| **Component** | **Theoretical concentration (ng/mL)** | **Mean concentration ± SD (ng/mL)** | **Precision, RSD (%)** | **Accuracy (%)** |
| --- | --- | --- | --- | --- |
| AFB1 | 0.50 | 0.47 ± 0.04 | 7.7 | -6.3 |
|  | 5.0 | 4.98 ± 0.19 | 3.7 | -0.4 |
|  | 50.0 | 50.49 ± 1.93 | 3.8 | 1.0 |
| AFB2 | 0.50 | 0.47 ± 0.47 | 10.0 | -7.0 |
|  | 5.0 | 5.34 ± 0.28 | 5.3 | 6.9 |
|  | 50.0 | 50.61 ± 1.77 | 3.5 | 1.2 |
| AFG1 | 0.50 | 0.60 ± 0.02 | 3.9 | 19.8 |
|  | 5.0 | 5.20 ± 0.14 | 2.7 | 4.1 |
|  | 50.0 | 50.10 ± 1.27 | 2.5 | 0.2 |
| AFG2 | 0.50 | 0.52 ± 0.03 | 5.6 | 3.9 |
|  | 5.0 | 5.14 ± 0.27 | 5.3 | 2.8 |
|  | 50.0 | 49.21 ± 1.12 | 2.3 | -1.6 |
| AFM1 | 0.50 | 0.50 ± 0.02 | 4.3 | -0.8 |
|  | 5.0 | 4.98 ± 0.40 | 8.0 | -0.5 |
|  | 50.0 | 50.68 ± 1.28 | 2.5 | 1.4 |
| AFM2 | 5.0 | 5.31 ± 0.14 | 2.6 | 6.1 |
|  | 50.0 | 56.79 ± 3.75 | 6.6 | 13.6 |
| AME | 0.50 | 0.49 ± 0.04 | 7.4 | -1.9 |
|  | 5.0 | 5.12 ± 0.11 | 2.1 | 2.5 |
|  | 50.0 | 50.98 ± 1.38 | 2.7 | 2.0 |
| AOH | 0.50 | 0.51 ± 0.01 | 2.2 | 2.1 |
|  | 5.0 | 5.03 ± 0.09 | 1.8 | 0.7 |
|  | 50.0 | 51.28 ± 1.09 | 2.1 | 2.6 |
| CIT | 0.50 | 0.47 ± 0.02 | 4.2 | -5.4 |
|  | 5.0 | 4.78 ± 0.26 | 5.3 | -4.5 |
|  | 50.0 | 47.02 ± 2.40 | 5.1 | -6.0 |
| DON | 5.0 | 4.47 ± 0.65 | 14.5 | -10.5 |
|  | 50.0 | 49.60 ± 1.96 | 4.0 | -0.8 |
| DOM-1 | 5.0 | 5.11 ± 0.46 | 8.9 | 2.3 |
|  | 50.0 | 53.20 ± 2.36 | 4.4 | 6.4 |
| 3-ADON | 5.0 | 5.53 ± 0.95 | 17.2 | 10.6 |
|  | 50.0 | 52.86 ± 1.73 | 3.3 | 5.7 |
| DON-GlcA | 5.0 | 5.02 ± 0.59 | 11.8 | 0.4 |
|  | 50.0 | 51.40 ± 1.89 | 3.7 | 2.8 |
| BEA | 0.50 | 0.54 ± 0.03 | 4.8 | 7.3 |
|  | 5.0 | 5.27 ± 0.25 | 4.8 | 5.4 |
|  | 50.0 | 55.79 ± 2.22 | 4.0 | 11.6 |
| ENNA | 0.50 | 0.54 ± 0.03 | 4.9 | 7.7 |
|  | 5.0 | 5.41 ± 0.23 | 4.2 | 8.2 |
|  | 50.0 | 52.37 ± 1.45 | 2.8 | 4.7 |
| ENNA1 | 0.50 | 0.51 ± 0.03 | 5.5 | 1.2 |
|  | 5.0 | 5.05 ± 0.19 | 3.7 | 1.0 |
|  | 50.0 | 52.90 ± 1.28 | 2.4 | 5.8 |
| ENNB | 0.50 | 0.52 ± 0.03 | 5.6 | 3.2 |
|  | 5.0 | 5.21 ± 0.23 | 4.3 | 4.2 |
|  | 50.0 | 54.77 ± 2.27 | 4.2 | 9.5 |
| ENNB1 | 0.50 | 0.48 ± 0.02 | 4.1 | -4.7 |
|  | 5.0 | 4.76 ± 0.06 | 1.3 | -4.9 |
|  | 50.0 | 48.60 ± 0.73 | 1.5 | -2.8 |
| FB1 | 0.50 | 0.59 ± 0.11 | 17.7 | 18.7 |
|  | 5.0 | 6.25 ± 0.85 | 13.5 | 25.0 |
|  | 50.0 | 61.05 ± 6.08 | 10.0 | 22.1 |
| OTA | 0.50 | 0.51 ± 0.02 | 3.9 | 1.0 |
|  | 5.0 | 5.10 ± 0.19 | 3.7 | 2.1 |
|  | 50.0 | 50.85 ± 1.63 | 3.2 | 1.7 |
| T2 | 5.0 | 5.06 ± 0.16 | 3.1 | 1.2 |
|  | 50.0 | 52.00 ± 1.82 | 3.5 | 4.0 |
| HT2 | 5.0 | 5.07 ± 1.43 | 28.2 | 1.3 |
|  | 50.0 | 51.38 ± 4.17 | 8.1 | 2.8 |
| TEA | 0.50 | 0.52 ± 0.08 | 15.6 | 3.3 |
|  | 5.0 | 4.87 ± 0.41 | 8.5 | -2.7 |
|  | 50.0 | 50.51 ± 0.95 | 1.9 | 1.0 |
| ZEN | 0.50 | 0.49 ± 0.07 | 14.3 | -2.7 |
|  | 5.0 | 5.40 ± 0.15 | 2.8 | 7.9 |
|  | 50.0 | 56.19 ± 1.02 | 1.8 | 12.4 |
| ZAN | 0.50 | 0.46 ± 0.04 | 9.5 | -7.3 |
|  | 5.0 | 5.17 ± 0.24 | 4.6 | 3.5 |
|  | 50.0 | 54.46 ± 2.73 | 5.0 | 8.9 |
| a-ZEL | 0.50 | 0.52 ± 0.12 | 23.5 | 3.3 |
|  | 5.0 | 5.07 ± 0.35 | 6.8 | 1.4 |
|  | 50.0 | 51.33 ± 2.32 | 4.5 | 2.7 |
| b-ZEL | 0.50 | 0.51 ± 0.05 | 9.4 | 2.8 |
|  | 5.0 | 5.49 ± 0.36 | 6.6 | 9.9 |
|  | 50.0 | 57.69 ± 2.34 | 4.1 | 15.4 |
| a-ZAL | 0.50 | 0.64 ± 0.11 | 17.5 | 27.8 |
|  | 5.0 | 5.47 ± 0.18 | 3.3 | 9.4 |
|  | 50.0 | 60.00 ± 6.40 | 10.7 | 20.0 |
| b-ZAL | 0.50 | 0.55 ± 0.04 | 6.3 | 9.6 |
|  | 5.0 | 5.42 ± 0.13 | 2.4 | 8.4 |
|  | 50.0 | 53.67 ± 1.48 | 2.8 | 7.3 |
| ZEN-Sulf | 50.0 | 46.08 ± 1.30 | 2.8 | -7.8 |
| Within-run accuracy and precision (n≥5); ^b^ Between-run accuracy and precision (n≥5, 3 analysis days); SD: standard deviation; RSD: relative standard deviation; Acceptance criteria: accuracy: < 1 ng/ml: -50% to+20%, ≥1 to <10 ng/ml: -40% to+20%, ≥ 10 -< 100 ng/ml: -30% to +10%, within-run precision (RSD_max_): < 1 ng/ml: 30 %, ≥ 1 to < 10 ng/ml: 25.0%, ≥ 10 to < 100 ng/ml: 15.0%; between-run precision: < 1 ng/ml : 45 %, ≥ 1 to < 10 ng/ml: 32.0%, ≥ 10 to < 100 ng/ml: 23% [VICH GL49]. | | | | |

**Table S5.** Results of the within-run and between-run precision and accuracy evaluation for the analysis of multiple mycotoxins in chicken egg white.

| **Component** | **Theoretical concentration (µg/kg)** | **Mean concentration ± SD (µg/kg)** | **Precision, RSD (%)** | **Accuracy (%)** |
| --- | --- | --- | --- | --- |
| AFB1 | 0.05 ^a^ | 0.05 ± 0.01 | 24.5 | -6.8 |
|  | 0.05 ^b^ | 0.05 ± 0.01 | 21.8 | -8.9 |
|  | 2.5 ^a^ | 2.51 ± 0.09 | 3.4 | 0.3 |
|  | 2.5^b^ | 2.58 ± 0.10 | 3.8 | 3.0 |
|  | 10.0 ^b^ | 9.81 ± 0.36 | 3.7 | -1.9 |
|  | 10.0 ^b^ | 10.22 ± 0.49 | 4.8 | 2.2 |
| AFB2 | 0.05 ^a^ | 0.06 ± 0.01 | 9.0 | 16.2 |
|  | 0.05 ^b^ | 0.06 ± 0.01 | 12.0 | 10.6 |
|  | 2.5 ^a^ | 2.57 ± 0.32 | 12.5 | 2.6 |
|  | 2.5^b^ | 2.68 ± 0.34 | 12.9 | 7.1 |
|  | 10.0 ^a^ | 9.59 ± 0.48 | 5.0 | -4.1 |
|  | 10.0 ^b^ | 10.00 ± 0.71 | 7.1 | -0.0 |
| AFG1 | 0.05 ^a^ | 0.06 ± 0.01 | 12.3 | 12.9 |
|  | 0.05 ^b^ | 0.06 ± 0.01 | 11.5 | 10.7 |
|  | 2.5 ^a^ | 2.58 ± 0.16 | 6.3 | 3.1 |
|  | 2.5^b^ | 2.62 ± 0.14 | 5.2 | 4.9 |
|  | 10.0 ^a^ | 10.10 ± 0.27 | 2.7 | 1.0 |
|  | 10.0 ^b^ | 10.10 ± 0.49 | 4.9 | 1.0 |
| AFG2 | 0.05 ^a^ | 0.05 ± 0.01 | 12.1 | -1.3 |
|  | 0.05 ^b^ | 0.05 ± 0.01 | 12.2 | 7.1 |
|  | 2.5 ^a^ | 2.58 ± 0.33 | 12.8 | 3.2 |
|  | 2.5^b^ | 2.70 ± 0.33 | 12.1 | 8.2 |
|  | 10.0 ^a^ | 9.96 ± 0.48 | 4.8 | -0.4 |
|  | 10.0 ^b^ | 10.0 ± 0.72 | 7.2 | 0.2 |
| AFM1 | 0.05 ^a^ | 0.06 ± 0.01 | 12.4 | 13.7 |
|  | 0.05 ^b^ | 0.05 ± 0.01 | 9.3 | 9.7 |
|  | 2.5 ^a^ | 2.68 ± 0.20 | 7.3 | 7.1 |
|  | 2.5^b^ | 2.65 ± 0.20 | 7.6 | 6.0 |
|  | 10.0 ^a^ | 10.31 ± 0.48 | 4.7 | 3.1 |
|  | 10.0 ^b^ | 10.24 ± 0.52 | 5.1 | 2.5 |
| AFM2 | 1.0 ^a^ | 0.78 ± 0.08 | 9.7 | -21.6 |
|  | 1.0 ^b^ | 0.87 ± 0.15 | 17.9 | -13.5 |
|  | 2.5 ^a^ | 2.53 ± 0.25 | 9.7 | 1.2 |
|  | 2.5^b^ | 2.41 ± 0.26 | 11.0 | -3.5 |
|  | 10.0 ^b^ | 9.94 ± 0.52 | 5.3 | -0.6 |
|  | 10.0 ^b^ | 9.58 ± 1.08 | 11.2 | -4.2 |
| AME | 0.05 ^a^ | 0.05 ± 0.01 | 4.8 | 0.3 |
|  | 0.05 ^b^ | 0.05 ± 0.00 | 4.4 | 1.8 |
|  | 2.5 ^a^ | 2.55 ± 0.09 | 3.4 | 2.1 |
|  | 2.5^b^ | 2.53 ± 0.09 | 3.7 | 1.1 |
|  | 10.0 ^a^ | 10.17 ± 0.21 | 2.0 | 1.7 |
|  | 10.0 ^b^ | 10.04 ± 0.22 | 2.2 | 0.4 |
| AOH | 0.50 ^a^ | 0.60 ± 0.06 | 10.2 | 20.5 |
|  | 0.50 ^b^ | 0.52 ± 0.08 | 15.7 | 3.2 |
|  | 2.5 ^a^ | 2.65 ± 0.05 | 2.0 | 6.1 |
|  | 2.5^b^ | 2.62 ± 0.18 | 6.9 | 4.7 |
|  | 10.0 ^a^ | 10.21 ± 0.32 | 3.1 | 2.1 |
|  | 10.0 ^b^ | 10.39 ± 0.93 | 9.0 | 3.9 |
| CIT | 0.25 ^a^ | 0.25 ± 0.01 | 1.7 | -0.9 |
|  | 0.25 ^b^ | 0.23 ± 0.03 | 11.3 | -8.0 |
|  | 2.5 ^a^ | 2.64 ± 0.04 | 1.5 | 5.5 |
|  | 2.5^b^ | 2.41 ± 0.22 | 9.1 | -3.6 |
|  | 10.0 ^a^ | 10.06 ± 0.17 | 1.7 | 0.6 |
|  | 10.0 ^b^ | 9.40 ± 0.76 | 8.1 | -6.0 |
| DON | 0.25 ^a^ | 0.24 ± 0.01 | 2.4 | -4.6 |
|  | 0.25 ^b^ | 0.24 ± 0.02 | 6.5 | -3.0 |
|  | 2.5 ^a^ | 2.60 ± 0.10 | 4.0 | 3.9 |
|  | 2.5^b^ | 2.52 ± 0.12 | 4.6 | 0.7 |
|  | 10.0 ^a^ | 10.62 ± 0.20 | 1.9 | 6.2 |
|  | 10.0 ^b^ | 10.43 ± 0.43 | 4.2 | 4.3 |
| DOM-1 | 0.50 ^a^ | 0.50 ± 0.03 | 5.8 | 0.4 |
|  | 0.50 ^b^ | 0.52 ± 0.08 | 15.1 | 3.6 |
|  | 2.5 ^a^ | 2.49 ± 0.05 | 2.1 | -0.5 |
|  | 2.5^b^ | 2.60 ± 0.23 | 8.9 | 4.0 |
|  | 10.0 ^a^ | 9.24 ± 0.37 | 4.0 | -7.6 |
|  | 10.0 ^b^ | 9.94 ± 0.74 | 7.5 | -0.6 |
| 3-ADON | 1.0 ^a^ | 1.04 ± 0.11 | 10.7 | 3.7 |
|  | 1.0^b^ | 1.03 ± 0.09 | 9.1 | 2.7 |
|  | 2.5 ^a^ | 2.57 ± 0.09 | 3.5 | 2.7 |
|  | 2.5^b^ | 2.65 ± 0.13 | 4.9 | 6.2 |
|  | 10.0 ^b^ | 9.81 ± 0.73 | 7.4 | -1.9 |
|  | 10.0 ^b^ | 10.15 ± 0.78 | 7.7 | 1.5 |
| DON-GlcA | 1.00 ^a^ | 1.06 ± 0.07 | 6.9 | 5.6 |
|  | 1.00 ^b^ | 1.02 ± 0.09 | 8.8 | 2.1 |
|  | 2.5 ^a^ | 2.46 ± 0.14 | 5.5 | -1.6 |
|  | 2.5^b^ | 2.44 ± 0.17 | 6.9 | -2.5 |
|  | 10.0 ^a^ | 10.58 ± 0.53 | 5.0 | 5.8 |
|  | 10.0 ^b^ | 10.23 ± 0.84 | 8.2 | 2.3 |
| BEA | 0.05 ^a^ | 0.03 ± 0.01 | 24.7 | -34.0 |
|  | 0.05 ^b^ | 0.04 ± 0.01 | 22.9 | -14.8 |
|  | 2.5 ^a^ | 2.59 ± 0.14 | 5.4 | 3.5 |
|  | 2.5^b^ | 2.28 ± 0.32 | 13.9 | -8.9 |
|  | 10.0 ^a^ | 10.38 ± 1.26 | 12.1 | 3.8 |
|  | 10.0 ^b^ | 9.61 ± 1.37 | 14.3 | -3.9 |
| ENNA | 0.05 ^a^ | 0.04 ± 0.01 | 18.7 | -24.8 |
|  | 0.05 ^b^ | 0.04 ± 0.01 | 15.2 | -17.4 |
|  | 2.5 ^a^ | 2.83 ± 0.03 | 1.0 | 13.1 |
|  | 2.5^b^ | 2.47 ± 0.30 | 12.1 | -1.1 |
|  | 10.0 ^a^ | 10.62 ± 0.79 | 7.4 | 6.2 |
|  | 10.0 ^b^ | 9.41 ± 1.36 | 14.4 | -5.9 |
| ENNA1 | 0.05 ^a^ | 0.05 ± 0.01 | 10.8 | -3.5 |
|  | 0.05 ^b^ | 0.05 ± 0.00 | 9.7 | -3.9 |
|  | 2.5 ^a^ | 2.55 ± 0.09 | 3.6 | 1.8 |
|  | 2.5^b^ | 2.30 ± 0.26 | 11.4 | -8.2 |
|  | 10.0 ^a^ | 9.71 ± 0.95 | 9.8 | -2.9 |
|  | 10.0 ^b^ | 9.13 ± 1.16 | 12.7 | -8.7 |
| ENNB | 0.05 ^a^ | 0.05 ± 0.01 | 12.0 | -1.9 |
|  | 0.05 ^b^ | 0.05 ± 0.01 | 11.9 | -3.9 |
|  | 2.5 ^a^ | 2.56 ± 0.09 | 3.6 | 2.2 |
|  | 2.5^b^ | 2.31 ± 0.26 | 11.2 | -7.5 |
|  | 10.0 ^a^ | 9.52 ± 1.01 | 10.6 | -4.8 |
|  | 10.0 ^b^ | 9.03 ± 1.03 | 11.4 | -9.7 |
| ENNB1 | 0.05 ^a^ | 0.04 ± 0.01 | 8.5 | -28.8 |
|  | 0.05 ^b^ | 0.04 ± 0.01 | 18.4 | -16.6 |
|  | 2.5 ^a^ | 1.81 ± 0.06 | 3.5 | -27.8 |
|  | 2.5^b^ | 1.97 ± 0.34 | 17.2 | -21.3 |
|  | 10.0 ^a^ | 9.49 ± 0.80 | 8.5 | -5.1 |
|  | 10.0 ^b^ | 7.82 ± 1.70 | 21.8 | -21.8 |
| FB1 | 0.25 ^a^ | 0.29 ± 0.06 | 19.7 | 14.0 |
|  | 0.25 ^b^ | 0.30 ± 0.07 | 23.5 | 21.5 |
|  | 2.5 ^a^ | 2.44 ± 0.14 | 5.6 | -2.3 |
|  | 2.5^b^ | 2.80 ± 0.64 | 22.8 | 12.2 |
|  | 10.0 ^a^ | 8.94 ± 0.56 | 6.3 | -10.6 |
|  | 10.0 ^b^ | 10.86 ± 2.41 | 22.2 | 8.6 |
| OTA | 0.05 ^a^ | 0.05 ± 0.01 | 4.2 | -1.3 |
|  | 0.05 ^b^ | 0.05 ± 0.00 | 4.8 | -1.1 |
|  | 2.5 ^a^ | 2.55 ± 0.07 | 2.8 | 2.1 |
|  | 2.5^b^ | 2.51 ± 0.08 | 3.3 | 0.3 |
|  | 10.0 ^a^ | 10.14 ± 0.14 | 1.3 | 1.4 |
|  | 10.0 ^b^ | 10.00 ± 0.23 | 2.3 | -0.0 |
| T2 | 1.0 ^a^ | 1.01 ± 0.08 | 7.5 | 0.8 |
|  | 1.0 ^b^ | 0.90 ± 0.22 | 24.4 | -9.8 |
|  | 2.5 ^a^ | 2.52 ± 0.15 | 5.8 | 0.7 |
|  | 2.5^b^ | 2.63 ± 0.21 | 8.0 | 5.3 |
|  | 10.0 ^a^ | 9.53 ± 0.68 | 7.1 | -4.7 |
|  | 10.0 ^b^ | 10.58 ± 1.10 | 9.9 | 5.8 |
| TEA | 1.0 ^a^ | 0.97 ± 0.04 | 4.0 | -3.5 |
|  | 1.0 ^b^ | 0.98 ± 0.05 | 5.2 | -1.7 |
|  | 2.5 ^a^ | 2.44 ± 0.12 | 4.7 | -2.3 |
|  | 2.5^b^ | 2.45 ± 0.13 | 5.4 | -2.0 |
|  | 10.0 ^a^ | 9.68 ± 0.54 | 5.5 | -3.2 |
|  | 10.0 ^b^ | 10.06 ± 0.73 | 7.3 | 0.6 |
| ZEN | 0.05 ^a^ | 0.05 ± 0.01 | 13.6 | 6.2 |
|  | 0.05 ^b^ | 0.05 ± 0.01 | 10.6 | 4.4 |
|  | 2.5 ^a^ | 2.57 ± 0.08 | 3.0 | 2.9 |
|  | 2.5^b^ | 2.59 ± 0.09 | 3.4 | 3.5 |
|  | 10.0 ^a^ | 10.28 ± 0.38 | 3.7 | 2.8 |
|  | 10.0 ^b^ | 10.26 ± 0.33 | 3.2 | 2.6 |
| ZAN | 0.05 ^a^ | 0.05 ± 0.01 | 4.3 | 7.6 |
|  | 0.05 ^b^ | 0.05 ± 0.01 | 11.1 | 8.2 |
|  | 2.5 ^a^ | 2.46 ± 0.12 | 5.0 | -1.8 |
|  | 2.5^b^ | 2.49 ± 0.16 | 6.5 | -0.3 |
|  | 10.0 ^a^ | 10.19 ± 0.64 | 6.2 | 1.9 |
|  | 10.0 ^b^ | 10.00 ± 0.47 | 4.6 | 0.0 |
| a-ZEL | 0.10 ^a^ | 0.08 ± 0.01 | 13.2 | -21.7 |
|  | 0.10 ^b^ | 0.09 ± 0.02 | 20.3 | -14.6 |
|  | 2.5 ^a^ | 2.49 ± 0.12 | 5.0 | -0.5 |
|  | 2.5^b^ | 2.41 ± 0.17 | 7.2 | -3.7 |
|  | 10.0 ^a^ | 10.18 ± 0.64 | 6.2 | 1.8 |
|  | 10.0 ^b^ | 10.04 ± 0.86 | 8.6 | 0.4 |
| b-ZEL | 0.25 ^a^ | 0.25 ± 0.02 | 7.5 | -0.6 |
|  | 0.25 ^b^ | 0.24 ± 0.02 | 7.3 | -3.2 |
|  | 2.5 ^a^ | 2.61 ± 0.13 | 4.8 | 4.2 |
|  | 2.5^b^ | 2.55 ± 0.17 | 6.8 | 2.0 |
|  | 10.0 ^a^ | 10.37 ± 0.53 | 5.2 | 3.7 |
|  | 10.0 ^b^ | 10.28 ± 0.93 | 9.0 | 2.8 |
| a-ZAL | 0.25 ^a^ | 0.26 ± 0.05 | 18.2 | 4.2 |
|  | 0.25 ^b^ | 0.24 ± 0.05 | 22.7 | -5.3 |
|  | 2.5 ^a^ | 2.67 ± 0.07 | 2.7 | 6.7 |
|  | 2.5^b^ | 2.48 ± 0.24 | 9.8 | -0.9 |
|  | 10.0 ^a^ | 10.76 ± 0.50 | 4.7 | 7.6 |
|  | 10.0 ^b^ | 10.33 ± 1.11 | 10.7 | 3.3 |
| b-ZAL | 0.05 ^a^ | 0.04 ± 0.01 | 22.6 | -16.9 |
|  | 0.05 ^b^ | 0.05 ± 0.01 | 17.4 | -4.6 |
|  | 2.5 ^a^ | 2.53 ± 0.15 | 6.0 | 1.4 |
|  | 2.5^b^ | 2.44 ± 0.22 | 8.8 | -2.3 |
|  | 10.0 ^a^ | 10.24 ± 0.45 | 4.4 | 2.4 |
|  | 10.0 ^b^ | 9.86 ± 0.93 | 9.4 | -1.4 |
| ZEN-Sulf | 0.25 ^a^ | 0.25 ± 0.01 | 3.0 | 0.0 |
|  | 0.25 ^b^ | 0.26 ± 0.04 | 14.8 | 4.8 |
|  | 2.5 ^a^ | 2.53 ± 0.10 | 3.8 | 1.2 |
|  | 2.5^b^ | 2.49 ± 0.41 | 16.5 | -0.4 |
|  | 10.0 ^a^ | 10.05 ± 0.81 | 8.1 | 0.5 |
|  | 10.0 ^b^ | 9.99 ± 1.17 | 11.7 | -0.1 |
| ^a^ Within-run accuracy and precision (n≥5); ^b^ Between-run accuracy and precision (n≥5, 3 analysis days); SD: standard deviation; RSD: relative standard deviation; Acceptance criteria: accuracy: < 1 µg/kg: -50% to +20%, ≥1 to <10 µg/kg: -40% to +20%, ≥ 10 - < 100 µg/kg: -30% to +10%, within-run precision (RSD_max_): < 1 µg/kg : 30 %, ≥ 1 to < 10 µg/kg: 25.0%, ≥ 10 to < 100 µg/kg: 15.0%; between-run precision: < 1 µg/kg : 45 %, ≥ 1 to < 10 µg/kg: 32.0%, ≥ 10 to < 100 µg/kg: 23% [VICH GL49]. | | | | |

**Table S6.** Evaluation of extraction recovery (RE), signal suppression/enhancement (SSE) and process efficiency (PE) for multiple mycotoxins in egg yolk and egg white.

| **Matrix** | **Egg Yolk** | | | **Egg White** | | |
| --- | --- | --- | --- | --- | --- | --- |
| **Component** | **RE (%)** | **SSE (%)** | **RA (%)** | **RE (%)** | **SSE (%)** | **RA (%)** |
| AFB1 | 73.0 | 57.1 | 41.7 | 50.4 | 61.0 | 30.8 |
| AFB2 | 82.5 | 70.4 | 58.1 | 54.2 | 55.4 | 30.0 |
| AFG1 | 83.2 | 69.5 | 57.8 | 55.9 | 55.1 | 30.8 |
| AFG2 | 84.1 | 65.8 | 55.4 | 57.3 | 48.0 | 27.5 |
| AFM1 | 80.0 | 64.2 | 51.4 | 59.7 | 55.7 | 33.2 |
| AFM2 | 81.6 | 63.8 | 52.0 | 78.1 | 51.6 | 40.3 |
| AME | 74.6 | 74.2 | 55.3 | 67.4 | 94.2 | 63.5 |
| AOH | 76.7 | 64.4 | 49.4 | 65.2 | 81.7 | 53.3 |
| CIT | 2.4 | 89.8 | 2.1 | 7.4 | 96.0 | 7.1 |
| DON | 68.1 | 29.4 | 20.0 | 64.0 | 20.4 | 13.1 |
| DOM-1 | 79.8 | 43.6 | 34.8 | 68.4 | 46.3 | 31.7 |
| 3-ADON | 82.3 | 46.3 | 38.1 | 67.7 | 54.2 | 36.7 |
| DON-GlcA | 16.8 | 27.0 | 4.5 | 53.2 | 10.0 | 5.3 |
| BEA | 82.0 | 52.5 | 43.0 | 63.4 | 83.5 | 52.9 |
| ENNA | 80.9 | 38.3 | 31.0 | 61.0 | 73.9 | 45.1 |
| ENNA1 | 78.8 | 65.1 | 51.3 | 63.0 | 86.1 | 54.2 |
| ENNB | 79.2 | 76.5 | 60.6 | 63.9 | 88.6 | 56.6 |
| ENNB1 | 78.4 | 71.8 | 56.3 | 64.2 | 84.2 | 54.0 |
| FB1 | 14.4 | 93.9 | 13.5 | 22.4 | 101.4 | 22.7 |
| OTA | 71.4 | 80.0 | 57.1 | 56.4 | 83.6 | 47.1 |
| T2 | 87.8 | 49.6 | 43.6 | 67.8 | 44.4 | 30.1 |
| TEA | 38.1 | 115.3 | 44.0 | 43.9 | 102.1 | 44.8 |
| ZEN | 80.2 | 72.2 | 58.0 | 69.7 | 89.3 | 62.3 |
| a-ZEL | 82.4 | 67.3 | 55.5 | 71.3 | 73.8 | 52.6 |
| b-ZEL | 80.5 | 58.2 | 46.9 | 67.0 | 80.9 | 54.2 |
| ZAN | 81.1 | 72.7 | 58.9 | 66.8 | 88.5 | 59.1 |
| a-ZAL | 80.0 | 69.1 | 55.3 | 69.7 | 83.0 | 57.9 |
| b-ZAL | 78.2 | 67.4 | 52.7 | 68.6 | 82.8 | 56.8 |
| ZEN-Sulf | 82.5 | 78.9 | 65.1 | 54.9 | 78.5 | 43.1 |

**Table S7.** Mean mycotoxin concentrations (± standard deviation, SD) in serum (n = 80), egg yolk (n = 160) and egg white (n = 160) of chickens that were reared at 38 poultry production farms in Ethiopia.

| **Mycotoxin** | **Mean^a^ Serum Conc. ± SD**  **(ng/ml)** | **Mean Egg Yolk Conc. ± SD**  **(µg/kg)** | **Mean Egg White Conc. ± SD**  **(µg/kg)** |
| --- | --- | --- | --- |
| AFB1 | < LOD^b^ | < LOD | < LOD |
| AFB2 | < LOD | < LOD | ND |
| AFG1 | < LOQ^c^ | < LOD | < LOD |
| AFG2 | < LOD | ND | ND |
| AFM1 | ND^d^ | ND | ND |
| AFM2 | < LOD | ND | ND |
| AME | < LOD | ND | < LOD |
| AOH | ND | ND | < LOD |
| CIT | < LOD | < LOD | < LOD |
| DON | ND | ND | < LOD |
| DOM-1 | < LOD | < LOD | < LOQ |
| 3-ADON | ND | ND | ND |
| DON-GlcA | ND | ND | ND |
| BEA | < LOD | < LOD | < LOD |
| ENNA | ND | ND | ND |
| ENNA1 | ND | ND | ND |
| ENNB | < LOQ | < LOQ | < LOD |
| ENNB1 | ND | < LOD | ND |
| FB1 | < LOD | ND | < LOD |
| OTA | 0.01 ± 0.12 | < LOD | ND |
| T2 | ND | < LOD | ND |
| HT2 | < LOD | NA | NA |
| TEA | 1.27 ±1.81 | < LOD | < LOD |
| ZEN | ND | < LOD | < LOD |
| a-ZEL | < LOQ | ND | ND |
| b-ZEL | ND | ND | ND |
| ZAN | < LOD | ND | < LOD |
| a-ZAL | ND | ND | ND |
| b-ZAL | ND | < LOD | < LOD |
| ZEN-Sulf | ND | < LOD | ND |
| Note: ^a^Mean concentrations: concentrations < LOQ were set to ½ LOQ and concentrations < LOD or ND were set to 0 ng/ml or µg/kg to calculate the mean concentrations per component; ^b^LOD = limit of detection, ^c^LOQ = limit of quantification; LOD and LOQ concentrations are reported in Table S2 (serum), Table 1 (egg yolk) and Table S3 (egg white); ^d^ND = not detected; NA = not applicable. | | | |

**Figure S1.** MRM chromatograms showing the quantifier ion trace for TEA and ^13^C_10_-TEA in a standard solution (concentration : 10 ng/ml) that was analyzed using (A) an Acquity Premier HSS-T3 (100 x 2.1 mm i.d., dp: 1.8 µm) and (B) an Acquity Premier BEH C18 (50 x 2.1 mm i.d., dp: 1.8 µm) column. The following chromatographic conditions were applied: mobile phase (MF) A: 0.1 % AA in water; MF B: 0.1 % AA in acetonitrile, flow-rate: 0.3 ml/min, 0 – 0.5 min (90% A, 10% B), 1.0 min (linear gradient to 35% B), 3.0 min (linear gradient to 60% B), 10 min (linear gradient to 95% B), 10 – 12.8 min (5% A, 95% B), 13.2 min (linear gradient to 10% B), 13.2 – 16.0 min (90% A, 10% B).

| (A) | (B) |
| --- | --- |
| 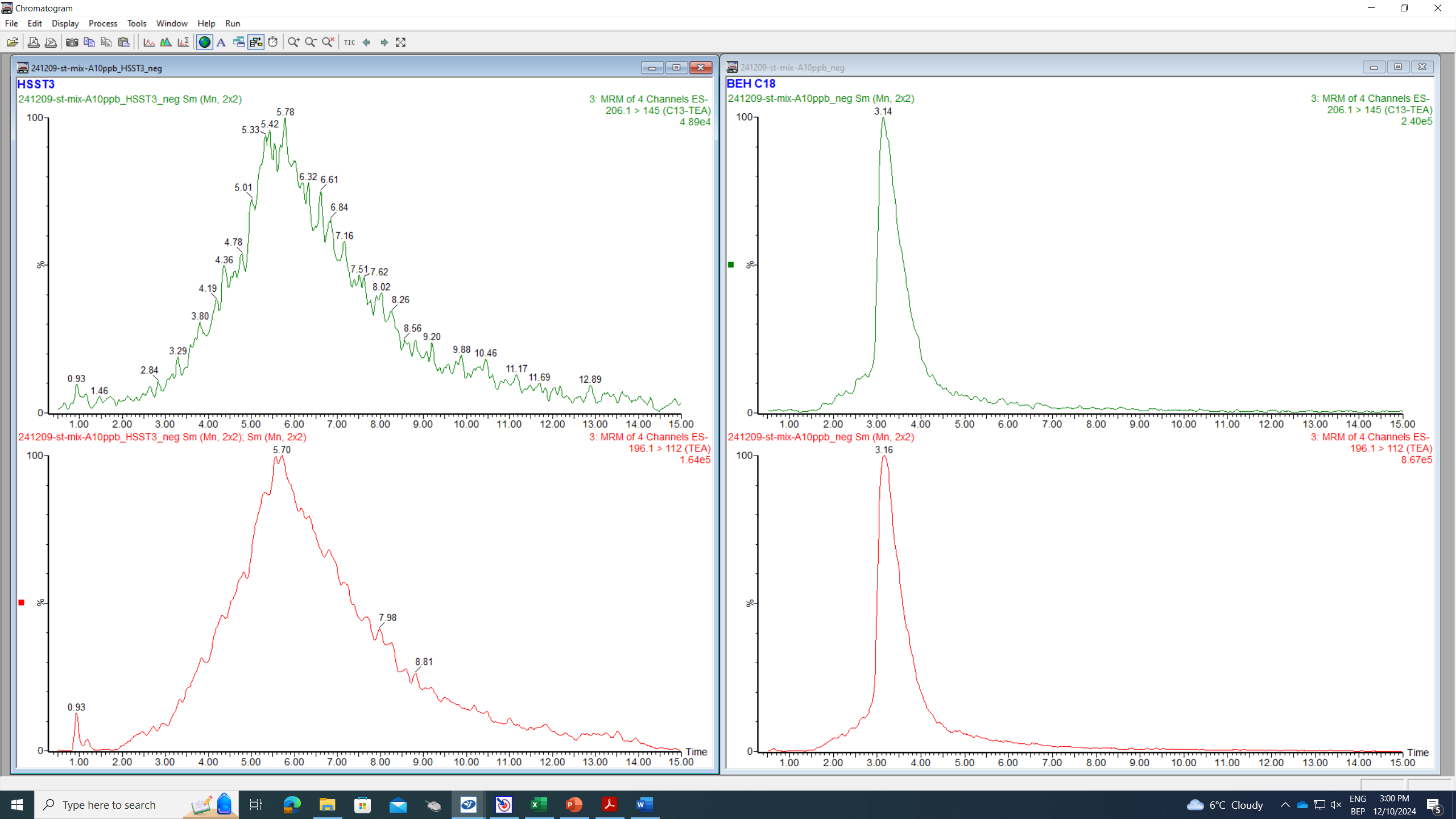 | 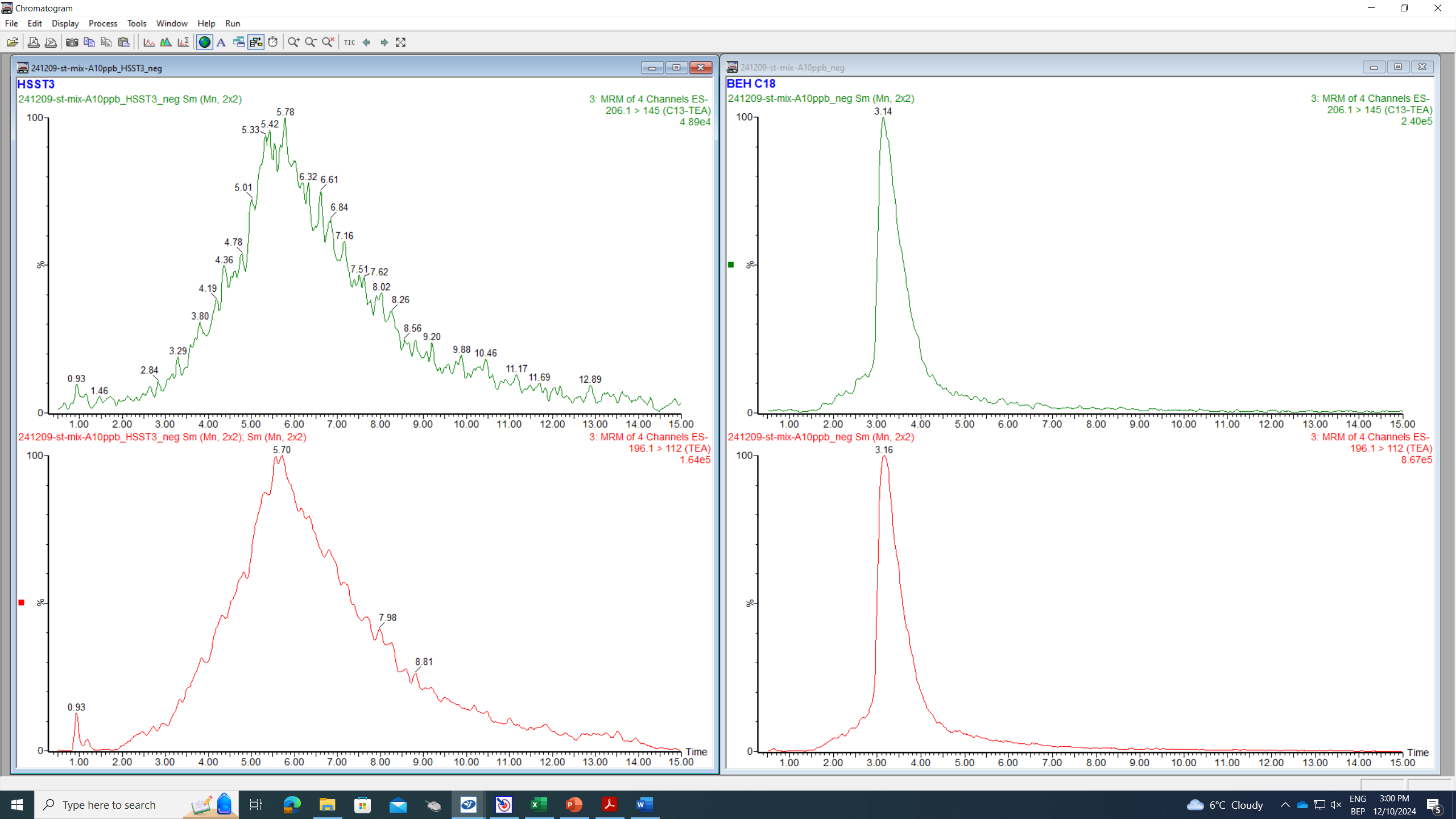 |
